# LEO1 Is Required for Efficient Entry into Quiescence, Control of H3K9 Methylation and Gene Expression in Human Fibroblasts

**DOI:** 10.3390/biom13111662

**Published:** 2023-11-17

**Authors:** Marc Laurent, Lina Cordeddu, Yasaman Zahedi, Karl Ekwall

**Affiliations:** Department of Biosciences and Nutrition, Neo Building, Karolinska Institutet, SE-141 83 Huddinge, Sweden; marclaurent1988@gmail.com (M.L.); lina.cordeddu@ki.se (L.C.); yasaman.zahedi@gmail.com (Y.Z.)

**Keywords:** cellular quiescence, PAF1C, LEO1, fibroblasts, chromatin, H3K9me2, histone modification, gene expression

## Abstract

(1) Background: The LEO1 (Left open reading frame 1) protein is a conserved subunit of the PAF1C complex (RNA polymerase II-associated factor 1 complex). PAF1C has well-established mechanistic functions in elongation of transcription and RNA processing. We previously showed, in fission yeast, that LEO1 controls histone H3K9 methylation levels by affecting the turnover of histone H3 in chromatin, and that it is essential for the proper regulation of gene expression during cellular quiescence. Human fibroblasts enter a reversible quiescence state upon serum deprivation in the growth media. Here we investigate the function of LEO1 in human fibroblasts. (2) Methods: We knocked out the *LEO1* gene using CRISPR/Cas9 methodology in human fibroblasts and verified that the LEO1 protein was undetectable by Western blot. We characterized the phenotype of the *ΔLEO1* knockout cells with FACS analysis and cell growth assays. We used RNA-sequencing using spike-in controls to measure gene expression and spike-in controlled ChIP-sequencing experiments to measure the histone modification H3K9me2 genome-wide. (3) Results: Gene expression levels are altered in quiescent cells, however factors controlling chromatin and gene expression changes in quiescent human cells are largely unknown. The *ΔLEO1* knockout fibroblasts are viable but have reduced metabolic activity compared to wild-type cells. *ΔLEO1* cells showed a slower entry into quiescence and a different morphology compared to wild-type cells. Gene expression was generally reduced in quiescent wild-type cells. The downregulated genes included genes involved in cell proliferation. A small number of genes were upregulated in quiescent wild-type cells including several genes involved in ERK1/ERK2 and Wnt signaling. In quiescent *ΔLEO1* cells, many genes were mis-regulated compared to wild-type cells. This included genes involved in Calcium ion transport and cell morphogenesis. Finally, spike-in controlled ChIP-sequencing experiments demonstrated that the histone modification H3K9me2 levels are globally increased in quiescent *ΔLEO1* cells. (4) Conclusions: Thus, LEO1 is important for proper entry into cellular quiescence, control of H3K9me2 levels, and gene expression in human fibroblasts.

## 1. Introduction

Dormant cells or quiescent cells are temporarily arrested in the cell cycle at the G_0_ stage. Quiescent cells can exit from G_0_ and return to the cell cycle in response to external signals or nutritional conditions. Therefore, quiescent cells have very important physiological functions [1]. For example, in humans, quiescent stem cells of the hemopoietic system can start proliferating in the bone marrow when new blood cells are needed in the body. Quiescent cells can also cause disease when quiescent cancer cells escape therapy and may cause relapse [2]. In unicellular eukaryotes, quiescent cells can survive starvation and allow the organism to be dormant until nutrients become available [3]. Thus, it is important to investigate how cells enter G_0_ and exit from G_0_ into proliferation. Also, we wish to understand how cells can maintain viability for a long time in the dormant state.

Human fibroblasts are an excellent model for studies of gene expression changes in quiescent cells. Fibroblasts enter the G_0_ state after 48 h of serum deprivation and pioneering microarray studies showed that this caused a massive change in gene expression [4]. A subsequent study showed that a common set of genes are regulated in quiescence induced by three independent signals—mitogen removal, contact inhibition, and loss of adhesion [5]. The study revealed that 116 genes were upregulated and 33 downregulated in quiescent cells induced by the three independent signals after 4 days. It has also been demonstrated that many mRNAs are regulated post-transcriptionally in quiescent fibroblasts. As many as 500 genes showed changes in mRNA decay rates in quiescence [6]. Regarding transcription factors the basic helix-loop-helix HES1 (Hairy and Enhancer of Split-1) has been shown to be a key factor for exit from quiescence [7].

Regarding chromatin structure and modification changes in quiescent cells, a few studies have been conducted. It was shown in human fibroblast that chromosome territories are changed upon cell cycle exit into G_0_ upon serum starvation so that a gene-poor chromosome is moved from the nuclear periphery to a more central position [8]. Single nucleosome imaging experiments in serum-starved G_0_ arrested human epithelial cells revealed that chromatin mobility is less restricted in the G_0_ cells, similar to cells treated with RNA pol II inhibitors or UV light [9]. Histone modification changes have been reported for serum-starved bovine fibroblasts which show a global reduction of H3K9me2 and H3K9me3 levels in quiescence [10]. Another histone modification, H4K20me3, is increased human fibroblasts during quiescence induced by in-contact inhibition [11]. Thus, chromatin structure changes and histone modifications are altered in mammalian G_0_ cells.

Our laboratory is particularly interested in chromatin and gene expression changes in quiescent cells. We and others have used genetic approaches in fission yeast cells to define factors required for survival in quiescence induced by nitrogen starvation. PAF1C is a conserved protein complex in eukaryotes with well-established mechanistic functions in the elongation of transcription and RNA processing [12]. Earlier we demonstrated the role of PAF1C in regulating H3K9 methylation levels in fission yeast [13]. Our genetic approach revealed that the PAF1C complex is important for maintaining viability during quiescence in fission yeast [14,15]. Fission yeast cells carrying a gene deletion for the gene encoding the PAF1C subunit LEO1 show a marked reduction of viability after 2–3 weeks in quiescence whereas wild-type control cells can survive for at least 4–5 weeks. In wild-type fission yeast cells H3K9 methylation levels i.e., H3K9me2 and H3K9me3 are reduced in quiescence [14]. In contrast, the *ΔLEO1* mutant cells show a reduced turnover of histone H3 in chromatin and increased H3K9me2 and H3K9me3 levels during quiescence compared to the wild type. The consequence of this histone modification change is a failure to properly regulate the expression of genes needed for survival in quiescence.

Here, we addressed the function of LEO1 in human fibroblasts subjected to quiescence and G_0_ arrest by serum deprivation. We show that LEO1 is required for efficient quiescence entry in human cells. Furthermore, using spike-in controlled RNA-seq and ChIP-seq experiments we show that LEO1 has an important role in regulation of gene expression and keeping H3K9me2 levels low in quiescent cells.

## 2. Materials and Methods

### 2.1. Cell Culture

hTERT-immortalized fibroblasts, BJ-5ta, were obtained from the American Type Culture Collection (ATCC; www.atcc.org). Cells were cultured in RPMI medium 1640 (Thermo Scientific, Stockholm, Sweden). The RPMI was supplemented with 10% FBS (Thermo Scientific, Stockholm, Sweden) and 1% penicillin/streptomycin (10,000 U/mL) (Thermo Scientific, Stockholm, Sweden). Cells were routinely passaged when the confluency reached 0.7–1 × 10^6^ cells/mL. Quiescent cells were obtained in cultures in RPMI medium 1640 supplemented with 0.1% FBS after ten days of incubation.

### 2.2. Cell Growth and Viability Assays

The cell growth assay was performed using the Cell proliferation Kit (product number 114650070001, Roche Solna, Sweden) according to the manufacturer’s instructions. Cells were incubated with the yellow tetrazolium salt (MTT) for 4 h. After the incubation period the salt crystals were solubilized by adding a solubilization buffer and the cells were incubated overnight at +37 °C, 5 to 6.5% CO_2_. The solubilized formazan product was spectrophotometrically quantified in quadruplicate samples using an ELISA reader at 570 nm.

### 2.3. FACS Analysis

Cultured cells (0.7–1 × 10^6^ cells/mL) were washed and resuspended in a prewarmed medium to reach 1 × 10^6^ cells/mL. Cells were stained with Hoechst dye using a final concentration of 2.5 μg/mL for proliferating cells and 1.5 μg/mL for quiescent cells. Cells were incubated with the dye for 30 min. at 37 °C for non-quiescence cells and 40 min at 37 °C for quiescent cells at the time points 24 h, 3 days, and 6 days, and for 30 min at 9 days. After incubation, cells were stained with Pyronin Y at a final concentration of 1.5 μg/mL, mixed, and incubated for 45 min at 37 °C. Then the cells were chilled on ice and protected from light. Before running the samples with a flow cytometer, cells were washed once with cold PBS containing 3% FCS, resuspended in cold PBS, and kept at 4 °C. The Cytoflex machine was used to analyze the cells. Based on their reduced RNA and DNA contents, G_0_ arrested cells were identified as populations with lower Hoechst and Pyronin Y signals.

### 2.4. Knockout of the LEO1 Gene

We used BJ-5ta cells to knock out the *LEO1* gene with CRISPR methodology. Two CRISPR plasmids from the CRISPR Nuclease Vector Kit (GeneArt^®^, Thermo Scientific, Stockholm, Sweden) were used to target the exons 1 and 2 of the *LEO1* gene were constructed. The following guide RNA’s were used: LEO1-gRNA-T1-fwd_TTTACGCTCAGCTTCGCTGTGTTTT; LEO1-gRNA-T1-rev_ACAGCGAAGCTGAGCGTAAACGGTG; LEO1-gRNA-T2-fwd_GACGAAGGTCATAGATCGGAGTTTT; LEO1-gRNA-T2-rev_TCCGATCTATGACCTTCGTCCGGTG. A mixture of the two plasmids (2.5 μg each) was electroporated in BJ-5ta cells using the NEON system (Thermo Fisher, Stockholm, Sweden). 24–48 h later, the cells were sorted by FACS for fluorescence signals coming from the plasmids. The cells were then subjected to single-cell cloning. The clones were tested by Western blot using the anti-LEO1 A300-175A antibody (BETHYL Laboratories, Montgomery, TX, USA) at 1/1000 dilution. The *ΔLEO1* clone (clone 13) was tested by PCR to confirm the loss of the DNA sequence between the exons 1 and 2 of the *LEO1* gene.

### 2.5. ChIP Sequencing

BJ-5ta fibroblasts were fixed in 1% paraformaldehyde for 8 min. Formaldehyde fixation was quenched by adding glycine (final concentration, 0.125 M) and ChIP was performed using the iDeal ChIP-seq kit (Diagenode, Liege, Belgium) according to the manufacturer’s instructions. Chromatin extracts from one million cells were sonicated to yield 100 to 500 bp fragments using a Bioruptor Pico sonication device (Diagenode, Liege, Belgium) set on high power for one round of 10 cycles with 30 s on and 30 s off at 4 °C. Fragmentation was verified after library preparation with agarose gel electrophoresis using an Agilent Tapestation 2200 machine and analysis software version 4.1.1 (Agilent, Sundbyberg, Sweden) to carry out a high sensitivity D1000 analysis (Supplementary Figure S3). Spike-in Chromatin (Active Motif, Waterloo, Belgium), was added in an amount of 10ng to each sample. ChIP-seq was performed using 1 μg of H3K9me2 antibody (ab1220, Abcam, Cambridge, UK) and 1 μg of spike-in antibody (anti-H2Av, Active Motif) per ChIP. After reverse cross-linking overnight at 65 °C, DNA was extracted using the Qiagen MinElute PCR purification kit in 15 μL of elution buffer. Libraries were prepared for sequencing using the NEBNext^®^ ChIP-Seq Library Prep Reagent Set for Illumina^®^ and sequenced on a HiSeq 2500 System (Illumina, San Diego, CA, USA).

### 2.6. RNA Sequencing

Total RNA was subjected to quality control with Agilent Tapestation according to the manufacturer’s instructions. ERCC RNA Spike-in Mix 1:1000 dilution was used for each sample. To construct libraries suitable for Illumina sequencing, the Illumina stranded mRNA prep ligation sample preparation protocol was used with a starting concentration between 25–1000 ng total RNA. The protocol includes mRNA isolation, cDNA synthesis, ligation of adapters, and amplification of indexed libraries. The yield and quality of the amplified libraries were analyzed using Qubit by Thermo Fisher and quality was checked by using Agilent Tapestation. The indexed cDNA libraries were normalized and combined, and the pools were sequenced on the Illumina Nextseq 2000 P2 100 cycle sequencing run, generating 58 bases paired-end reads with dual index 10 + 10 base pairs. Base-calling and demultiplexing were performed using CASAVA software version 1.8.2 with default settings generating Fastq files for further downstream mapping and analysis.

### 2.7. Bioinformatics and Statistical Analysis

Raw sequencing data from Nextseq 2000 (Bcl files) were converted and demultiplexed to fastq files using the bcl2fastq v2.20.0.422 program. The STAR 2.7.9a program [16] was used to index the reference genome and the ERCC spike-in sequences, and then the resulting fastq files were aligned. The mapped reads were then counted in annotated exons using featureCounts v1.5.1 [17]. The genome fasta file and annotations were obtained from the ensemble. The count table from ‘featureCounts’ was imported into the R/Bioconductor program and differential gene expression analysis was performed using the EdgeR package [18]. The resulting gene lists of differentially expressed genes after ERCC normalization are shown in the Supplementary Data. The linear model’s pipeline of EdgeR was used. For the gene expression analysis, genes that had >1 count per million in 3 or more samples were used and normalized based only on the ERCC spike-in counts using the TMM normalization. For the ChIP-seq we used the csaw R package to count the ChIP signals genome-wide and then we normalized to Spike-in Chromatin controls.

## 3. Results

### 3.1. Knock out of the LEO1 Gene 

**Figure 1:**
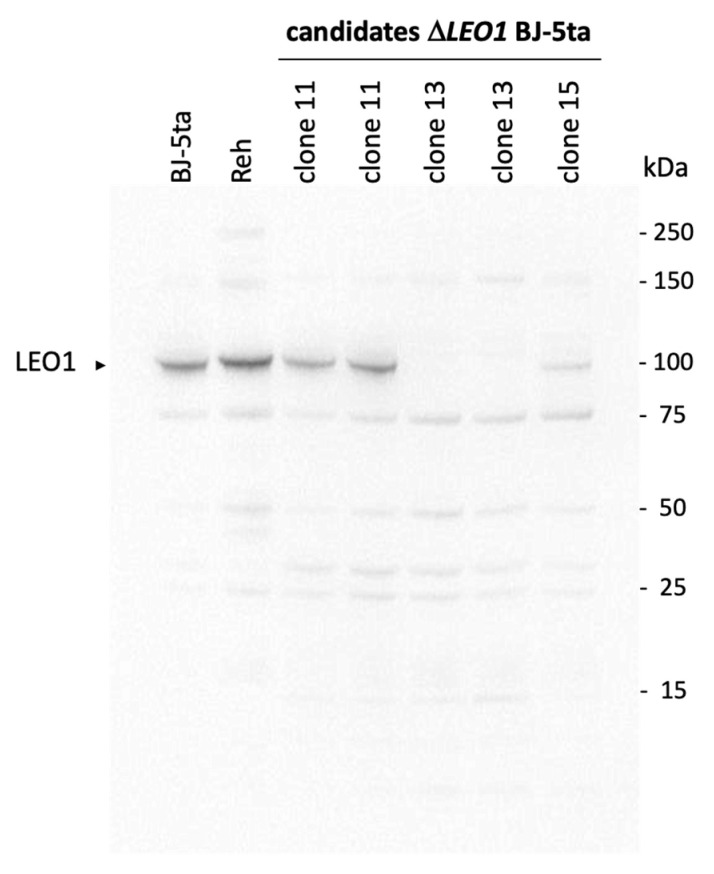
Confirmation of CRISPR-Cas9-mediated LEO1 knockout. The LEO1 knockout cell line was generated by CRISPR-Cas9 in Bj-5ta fibroblasts and confirmed by immunoblotting. Three candidates were chosen to validate the knockout and clone 13 was selected for further experiments in this study. The Reh cell line was included as a positive control since it overexpresses LEO1. Original western blot can be found in Figure S1.

We used CRISPR/Cas9 methodology to knock out the first two exons of *LEO1* gene in human Bj-5ta fibroblasts (See Materials Methods). We then verified LEO1 protein expression in the knock-out cell lines by Western blotting (Figure 1). It was clear that LEO1 protein expression was undetectable in clone 13 so we used this *ΔLEO1* clone for all the subsequent analysis (Figure 1).

Next, we performed a tetrazolium dye (MTT) assay for the metabolic activity of *ΔLEO1* cells (clone 13) and wild-type control cells (Supplementary Figure S2A). Cells were incubated with MTT, and *ΔLEO1* cells reduced this dye at levels of 65% rate compared to wild type in 10% serum, and at 30% compared to wild type in 0.1% serum. These differences were highly significant (2-sided *t*-test *p* < 0.002; n = 4) suggesting that the metabolic activity of *ΔLEO1* cells is somehow inhibited compared to wild type, especially in conditions of serum deprivation.

### 3.2. A Phenotype for ΔLEO1 Cells in Quiescence

**Figure 2:**
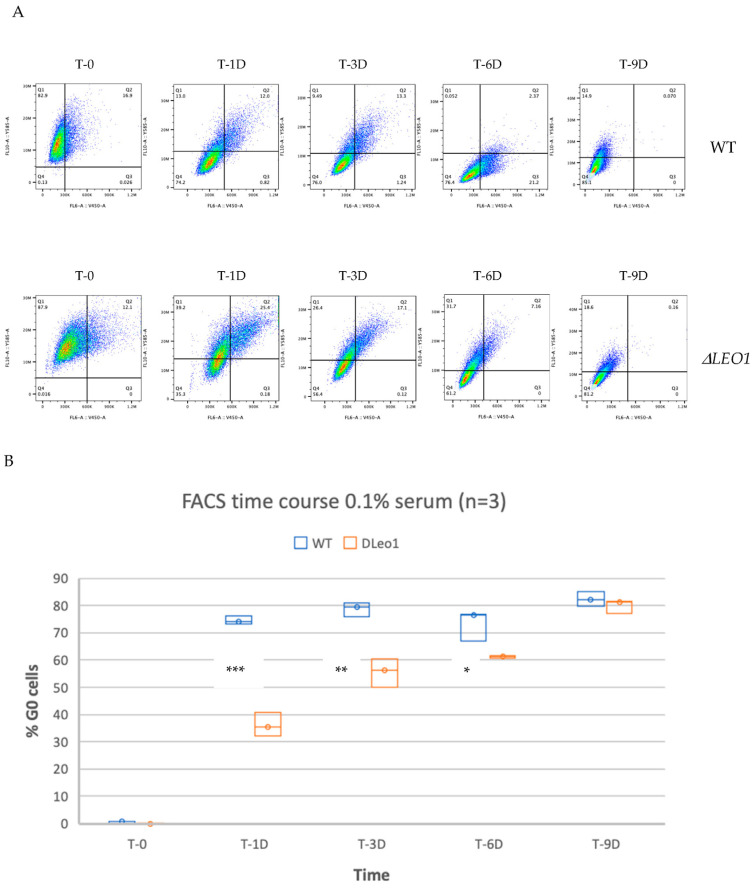
Slow entry of *ΔLEO1* cells in quiescence. Cells were stained with Hoechst to detect DNA and Pyronin Y to detect RNA. (**A**) FACS analysis of wild type (top panel) and *ΔLEO1* cells (bottom panel). The RNA content is shown on the Y-axis and the DNA content is shown on the X-axis. The G_0_ arrested cells are scored in the Q4 region of the diagram. (**B**) Quantitation of quiescent (G_0_ arrested) cells from the FACS time course experiment represented as a box plot. In each box, the median value is indicated by the horizontal black line inside the box. The top and the bottom of the box are the 75 and 25 percentiles respectively. Significant changes are indicated by * (two-sided *t*-test; *p* < 0.02) ** (2-sided *t*-test; *p* < 0.01) and *** (two-sided *t*-test; *p* < 0.001).

Next, we examined the cellular phenotype of *ΔLEO1* and wild-type cells in more detail by FACS analysis. We used a double gating strategy to measure the DNA content with Hoechst staining and the RNA content with Pyronin Y labeling. Quiescent cells were recognized by their reduced RNA content and a 1C DNA content indicative of G_0_ arrest. Both the *ΔLEO1* and wild-type cells were arrested in G_0_ after 9 days of incubation in growth media with 0.1% serum (Figure 2A).

**Figure 3:**
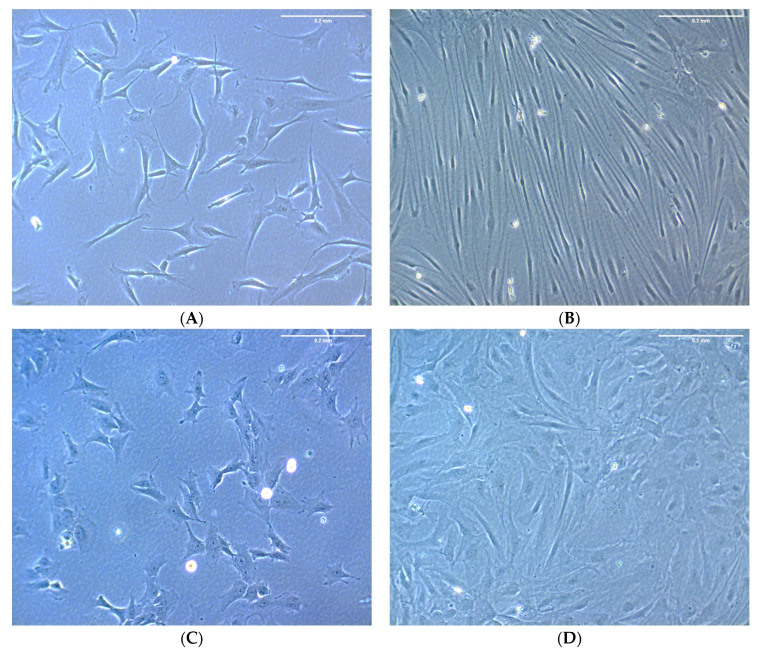
Bj-5ta wild type and *ΔLEO1* morphology were examined under a light-inverted microscope. (**A**,**C**) Cell morphology was observed after culturing Bj-5ta wild type (**A**) and *ΔLEO1* (**C**) in a medium with 10% serum (before shift to 0.1% serum). (**B**,**D**) Cell morphology was observed after incubating Bj-5ta wild type (**B**) and *ΔLEO1* (**D**) in a medium with 0.1% serum ten days after the shift. Size bar = 0.2 mm.

By quantifying the number of G_0_ cells over time it was obvious that the *ΔLEO1* cells showed a slower entry into quiescence compared to wild-type cells (Figure 2B). Interestingly, *ΔLEO1* cells in G_0_ also showed a different morphology compared to the wild type. The wild-type cells became uniformly elongated after 10 days in low serum, whereas the *ΔLEO1* cells were still irregular in shape (Figure 3). To quantify this phenotypic change we measured the cell length in a time course experiment upon a shift from 10% to 0.1% serum and found a significant difference at all time points (ANOVA *p*-value < 2.4 × 10^−4^). The average length of *ΔLEO1* cells did not exceed 151 μm whereas wt cells reached an average length of 283 μm (Supplementary Figure S2C). These observations indicated that *ΔLEO1* cells experienced some time delay in quiescence entry and displayed an aberrant phenotype when they eventually arrested in G_0_.

### 3.3. Changes in Gene Expression

Wild-type cells entering quiescence have a strongly reduced RNA content. Therefore, we used synthetic External RNA Controls Consortium (ERCC) spike-in controls in our RNA-sequencing experiments to allow for measurements of global changes in gene expression. We employed a statistical cut-off to define significantly downregulated and upregulated genes in quiescent cells compared to proliferating cells. This analysis revealed that as many as 5350 genes were downregulated and only 162 genes were upregulated in quiescent wild-type cells.

**Figure 4:**
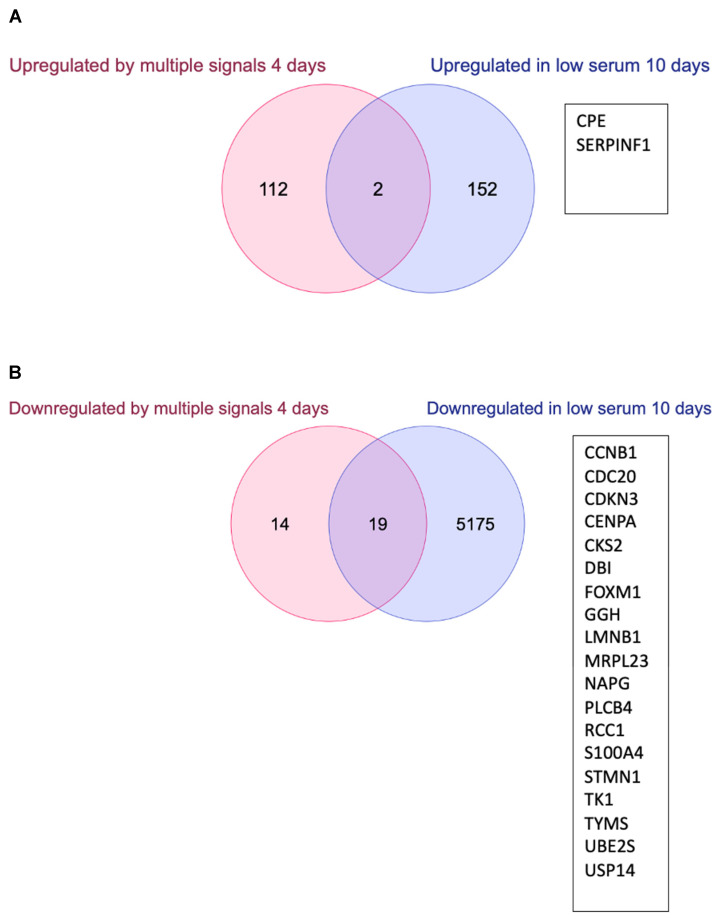
Gene expression changes in serum-starved cells (wt cells grown in 0.1% serum for 10 days compared to wt cells grown in 10% serum) and comparison to quiescence genes regulated by multiple signals after 4 days. Venn diagrams comparing previously defined genes upregulated (**A**) and downregulated (**B**) in quiescence induced by multiple signals after 4 days and low serum conditions after 10 days identified by this study. Gene names for genes in the intersections are shown.

Next, we compared lists of genes in the previously defined quiescence program [5] with our lists of upregulated and downregulated genes in 0.1% serum. This comparison identified only two commonly upregulated quiescence genes (Figure 4A).

CPE encodes carboxypeptidase E implicated in cell communication, signal transduction, and regulation of transcription. SERPINF1 encodes the serine proteinase inhibitor pigment epithelium-derived factor. The comparison of downregulated genes revealed a significant fraction of 19 genes in common for the different experiments (Figure 4B; hypergeometric distribution *p*-value = 0.00208). Regarding the downregulated genes GGH and USP14 are involved in amino acid metabolism and protein catabolism. CCNB1, CDC20, CDKN3, and CKS2 are cell cycle genes. CKS2 is the Cdc28 kinase regulatory subunit required for the G2/M transition. The UBE2S gene encodes a ubiquitin-conjugating enzyme essential for anaphase-promoting complex (APC) activity. CENPA is a centromere protein required for chromosome segregation. LMNB1 encodes lamin B1 and is involved in chromosome organization in the nucleus. TK1 (Thymidine kinase 1) and TYMS (Thymidylate synthetase) are required for nucleic acid metabolism and DNA replication in the S phase. RCC1. (Regulation of Chromosome Condensation protein 1) is involved in the G1/S transition. S100A4 is the gene for a calcium-binding protein. STMN1 and PLCB4 encode stathmin1 and phospholipase C, beta 4 respectively, both are implicated in cell communication. NAPG encodes the NSF Attachment Protein Gamma. MRPL23 is a mitochondrial ribosomal protein and DBI is a Diazepam binding inhibitor. Finally, FOXM1 is the gene for the Forkhead box M1 transcription factor protein. Thus, genes involved in cell cycle, cell division, DNA replication, metabolic functions, and some aspects of chromosome organization were found to be repressed in quiescence induced by three different stimuli after 4 days and in our experiment with serum starvation for 10 days.

To further examine the functions of genes affected by 10 days of serum deprivation in our experiment we performed a gene ontology (GO) analysis. This corroborated that the downregulated genes were involved in the cell division cycle, for example, 22 genes were required for mitotic spindle organization (1.15E^−03^) and 13 genes were involved in the initiation of DNA replication (*p* = 4.04E^−03^) (Figure 6A; Supplementary Table S1).

Several other biological processes were revealed by the GO analysis. The most enriched GO term for downregulated genes was double-stranded DNA repair via break-induced replication (fold enrichment 3.92; *p* = 2.52E^−03^).

Regarding the set of 162 upregulated genes there were many strongly enriched GO terms related to cell differentiation and cell signaling (See Figure 6B; Supplementary Table S2). For example, we found that two upregulated genes were involved in oligodendrocyte differentiation (fold enrichment 48.33; *p* = 1.27E^−03^) and three genes in regulating angiogenesis (fold enrichment 22.89; *p* = 4.40E^−04^). Regarding cell signaling five upregulated genes were involved in ERK1/ERK2 signaling cascade (fold enrichment 14.80; *p* = 3.39E^−05^), seven upregulated genes were implicated in the canonical Wnt (Wingless and Int-1) signaling pathway (fold enrichment 9.67; *p* = 1.21E^−05^) and eight upregulated genes were implicated in cell surface signaling pathways (fold enrichment 8.79; *p* = 5.55E^−06^). Several other GO terms related to cell differentiation, signaling, and organismal development were also significant. Hence, our spike-in approach extends the catalog of cellular processes being affected by serum starvation.

### 3.4. Changes in Gene Expression in ΔLEO1 Cells

**Figure 5:**
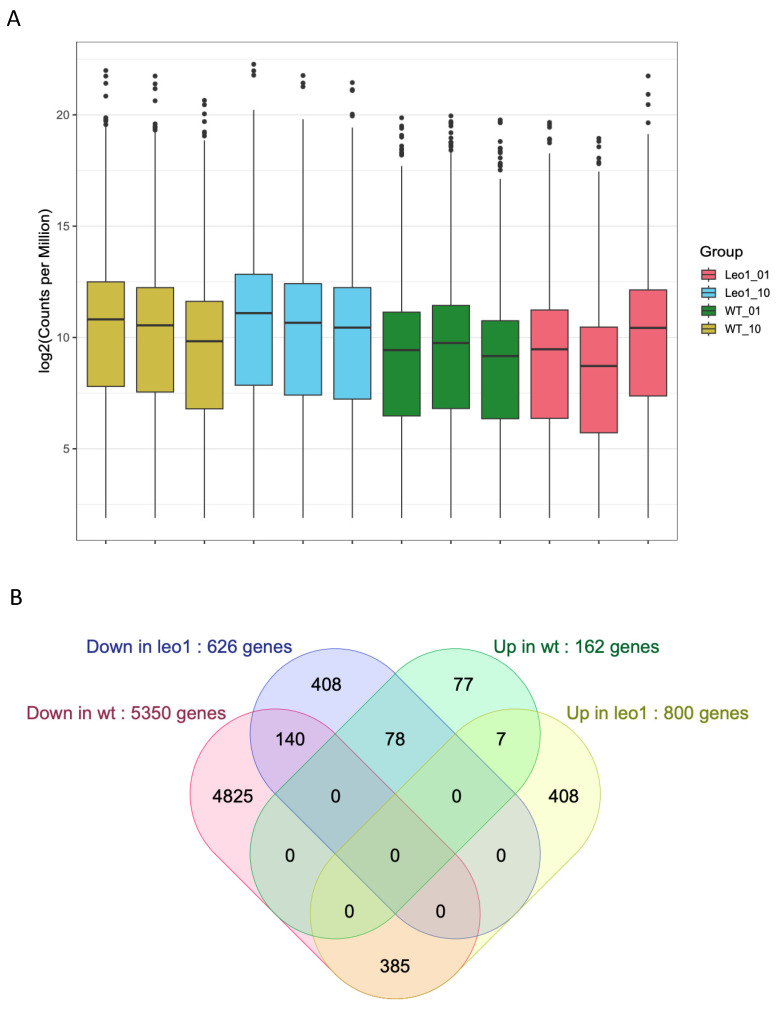
(**A**) ERCC spike-in normalized RNA-seq. The box plots show triplicate RNA-seq samples as log2 values of numbers of normalized sequence reads (*Y*-axis) in wild-type cells and in *ΔLEO1* cells cultivated in 10% or 0.1% serum as indicated (Leo1_01; Leo1_10; WT_01; WT_10). In each box, the median value is indicated by the horizontal black line inside the box. The top and the bottom of the box are the 75 and 25 percentiles respectively. The vertical line outside each box indicates the variation of the data and the dots are the outliers. (**B**) Venn diagrams comparing the list of downregulated (Down) and upregulated (Up) genes in wt (0.1% serum) compared to wt (10% serum) and in *ΔLEO1* cells grown in 0.1% serum compared to wt (0.1% serum).

Next, we compared the number of upregulated and downregulated genes between quiescent wild type and *ΔLEO1* cells using a statistical cut-off calculated from the triplicate spike-in normalized samples (Figure 5A). In *ΔLEO1* cells 626 genes were significantly downregulated and 800 genes were upregulated in 0.1% serum compared to 10% serum (Figure 5B). Compared to wild-type cells the overlap was small indicating a large number of misregulated genes in *ΔLEO1* cells.

**Figure 6:**
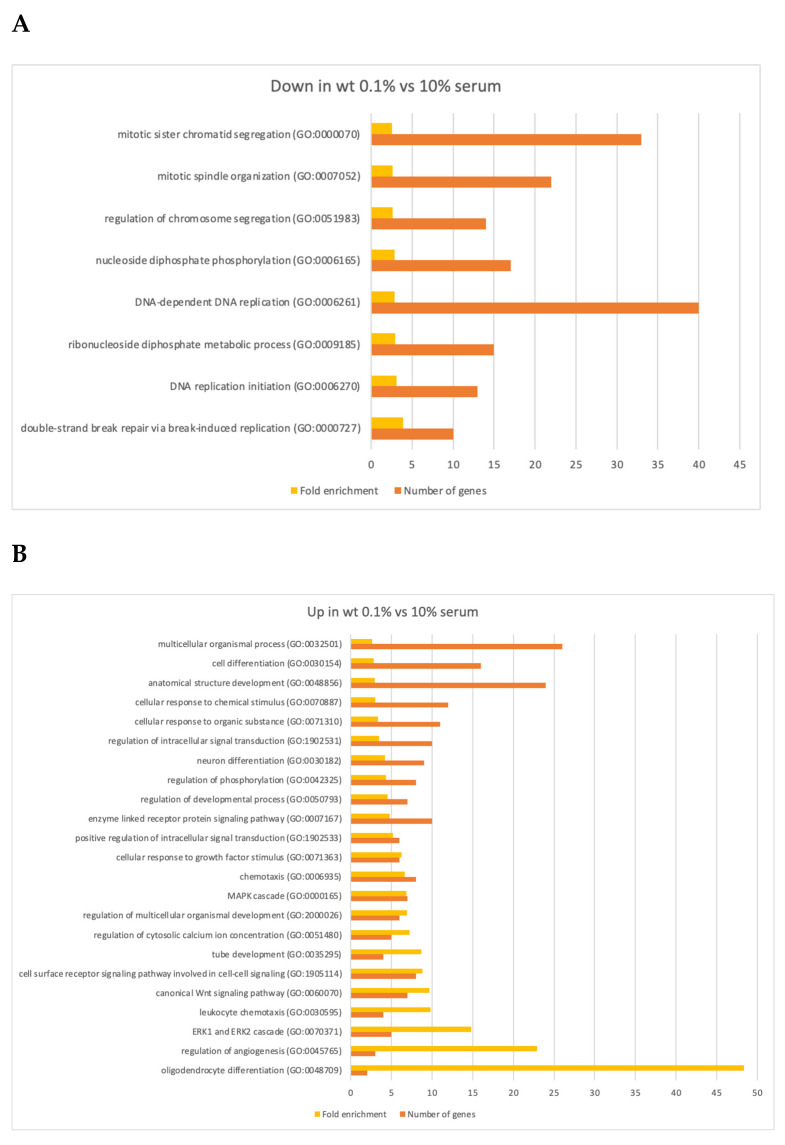

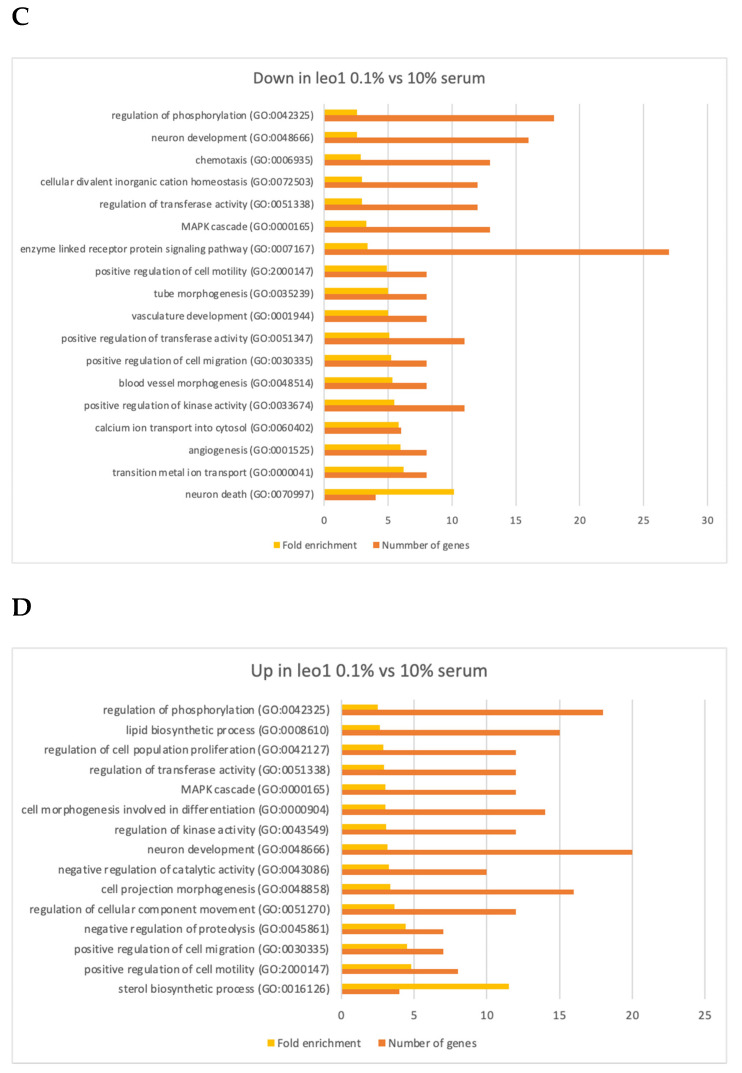
Summarized results from gene ontology analysis (PANTHER GO-Slim) of affected biological processes in RNA-seq experiment with wild-type cells and in *ΔLEO1* cells cultivated in 10% or 0.1% serum. (**A**) Significant GO terms for 5350 genes downregulated in wt (0.1% serum) vs. wt (10% serum). (**B**) Significant GO terms for 162 genes upregulated in wt (0.1% serum) vs. wt (10% serum). (**C**) Significant GO terms for 626 genes downregulated in *ΔLEO1* (0.1% serum) vs. *ΔLEO1* (10% serum). (**D**) Significant GO terms for 800 genes upregulated in *ΔLEO1* (0.1% serum) vs. *ΔLEO1* (10% serum). (**A**–**D**) Redundant GO terms have been omitted. Minimum fold enrichment = 2.5. The complete GO analysis results are shown in Supplementary Data.

Regarding the most highly enriched GO terms for the 626 downregulated genes in *ΔLEO1* in 0.1% serum compared to 10% serum we noticed the processes neuron death (4 genes; fold enrichment 10.13; *p* = 1.22E^−03^) transition metal ion transport (8 genes; fold enrichment 6.20; *p* = 1.21E^−05^) and calcium ion transmembrane transport into the cytosol (6 genes; fold enrichment 6.00; *p* = 8.54E^−04^) and angiogenesis (8 genes; fold enrichment 5.96; *p* = 1.25E^−04^) (Figure 6C, Supplementary Table S3). Several other processes all shown in Figure 6C were also significant; notably, eight of the downregulated genes were involved in cell migration (*p* = 2.77E^−04^) and 27 genes were implicated in enzyme-linked receptor protein signaling (*p* = 1.31E^−07^).

Regarding the 800 upregulated genes in *ΔLEO1* cells in 0.1% serum compared to 10% serum the most enriched GO terms were sterol biosynthetic process (4 genes; fold enrichment 11.50; *p* = 8.29E^−04^), positive regulation of cell motility (8 genes; fold enrichment 4.82; *p* = 4.62E^−04^), and positive regulation of cell migration (7 genes; fold enrichment 4.51; *p* = 1.48E^−03^) (Figure 6D; Supplementary Table S4). We also noticed several other enriched GO terms related to cell morphogenesis, differentiation, signaling, and cell proliferation. Thus, a significant number of genes involved in these processes were clearly mis-regulated in *ΔLEO1* cells.

### 3.5. Epigenome Changes in ΔLEO1 Cells

**Figure 7:**
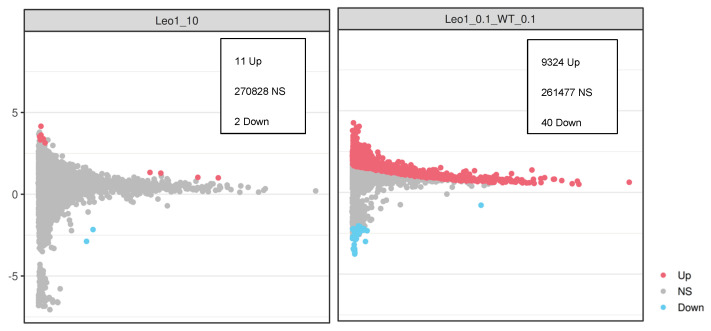
Chip-seq analysis of H3K9me2 levels in proliferating and quiescent cells. Moving average plots showing the significant changes of H3K9me2 detected in different comparisons. A 200 bp sliding window was used to count the number of reads every 50 bp across the whole genome. Merging windows that are overlapping to a single region. The numbers indicate regions found significant after multiple hypothesis testing (FDR). Regions were judged as significant if the adjusted *p*-value was under 0.05. The number of regions with increased levels (Up) nonsignificant (NS) or reduced levels (Down) are indicated. The data was normalized using the *Drosophila* spike-in procedure. The log fold change is shown on the *Y* axis and the log CPM is shown on the *X*-axis. (**left**) Leo1_10 indicates *ΔLEO1* cells compared to WT cells both grown in 10% serum; (**right**) Leo1_0.1_WT_0.1 indicates *ΔLEO1* cells compared to WT cells both in 0.1% serum.

**Figure 8:**
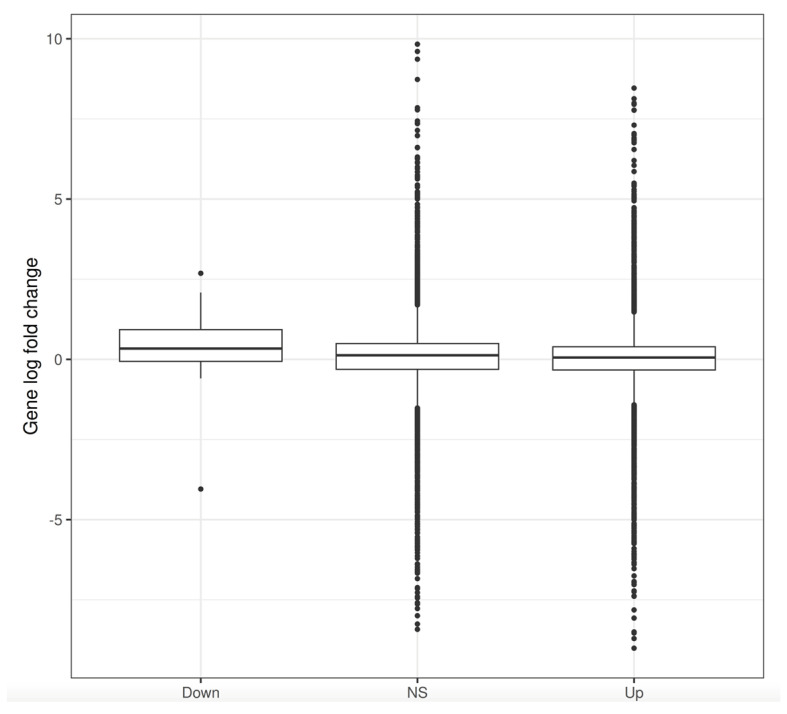
Box-plot comparisons of gene expression levels regions with significant H3K9me2 changes in *ΔLEO1* vs. WT. Gene expression changes in *ΔLEO1* vs. WT both grown in 0.1% serum, are shown on the *Y*-axis (Log fold change). H3K9me2 ChIP-seq reads were counted at all annotated TSS regions in a +/- 1 kb window. Regions were judged as significant if the adjusted *p*-value was under 0.05. The data was normalized using the *Drosophila* spike-in procedure. *X*-axis: 133 TSS regions had reduced levels (Down; *p* < 0.05), 198,626 regions were nonsignificant (NS) and 38,942 had increased levels (Up; *p* < 0.05).

Next, we investigated histone modification changes by ChIP-seq i.e., methylation of histone H3 at lysine 9 (H3K9me2). It was clear that H3K9me2 is affected by *ΔLEO1,* especially in conditions of low serum where 9324 genomic regions had H3K9me2 levels significantly increased and 40 reduced as compared to WT (Figure 7). To test if there was any relationship between gene expression changes in the *ΔLEO1* mutant we compared gene expression fold changes in TSS regions with reduced, unchanged, or increased H3K9me2 by box-plot analysis (Figure 8). This analysis revealed that gene expression was upregulated in the relatively small number of TSS regions with reduced H3K9me2 levels in the *ΔLEO1* mutant. In contrast, the 38,942 TSS regions with significantly increased H3K9me2 levels in *ΔLEO1* showed a slightly lower median value for gene expression.

## 4. Discussion

### 4.1. A Role for LEO1 in Quiescent Fibroblasts

Here we investigate the function of the *LEO1* gene encoding a PAF1C subunit in quiescent human fibroblasts. The study was initiated based on our previous work on the *LEO1* gene in fission yeast cells, which is implicated in survival during quiescence, regulation of chromatin modification, and gene expression. Our initial experiments revealed a phenotype for *ΔLEO1* cells showing a delay in quiescence entry compared to wild type and different cell morphology in low serum conditions. These physical changes in the cell structure may be associated with the delay in cell cycle exit into G_0_ [1]. Thus, we expected to see aberrant gene expression and chromatin modification patterns *ΔLEO1* cells that could be causative for the observed cellular phenotype.

### 4.2. Gene Expression Changes in Quiescent Wild Type and ΔLEO1 Cells

A pioneering genome-wide study used microarrays to identify genes regulated by serum deprivation of human fibroblasts in culture [4]. Subsequent microarray studies identified genes in the ‘quiescence program’ of fibroblasts using three independent signals that induce quiescence [5]. Here we used an RNA-sequencing approach with RNA spike-in controls to identify additional genes that respond to serum deprivation in human fibroblasts. Our approach also allowed us to reveal as many as 5350 downregulated genes in wild-type cells after 10 days in low serum. 19 of these genes were previously described as genes downregulated by multiple signals after 4 days [5]. The LMNB1 gene encoding Lamin B1 was in this group suggesting that the nuclear organization is changed in quiescent cells by altering chromosome contacts with the nuclear lamina. Changes in Lamin A/C localization have been observed in quiescent myoblasts [19]. It remains to be tested what the functional consequences are of Lamin B1 reorganization in quiescent cells. The GO analysis showed that several genes involved in processes coupled to cell division were downregulated in our experiment. This included four cell cycle genes (CCNB1, CDC20, CDKN3, and CKS2) previously shown to be repressed in quiescent fibroblasts [5]. Our analysis also identified 162 upregulated genes in low serum. These genes were enriched for several GO terms notably the processes of ERK1/ERK2 and Wnt signaling. Interestingly, Wnt signaling is known to enforce the quiescence of hematopoietic and neural stem cells [7,8].

Regarding gene expression in *ΔLEO1* cells, we found that many genes were misregulated in low serum compared to wild type (Figure 5B). Among the downregulated genes in *ΔLEO1* cells in 0.1% serum, several GO terms including ‘Calcium ion transport into cytosol’ were significant. The upregulated genes in *ΔLEO1* cells in 0.1% serum represented several GO terms related to cell morphology changes and cell migration. Although the exact mechanisms need to be elucidated, these misregulated genes likely contribute to the observed aberrant cellular phenotype of quiescent *ΔLEO1* cells. LEO1 could either directly affect gene expression via its interaction with RNA polymerase II or indirectly by affecting histone modification as was demonstrated in fission yeast [14]. The changes in gene expression and H3K9 methylation in *LEO1* cells could thus be two different aspects of PAF1C function.

### 4.3. Histone Modification Change of H3K9me2—Possible Mechanism

Both in bovine fibroblasts and fission yeast H3K9me2 and H3K9me3 levels are reduced during quiescence [10,14]. In these G_0_ arrested cells, there is no DNA replication occurring which is normally linked to histone turnover and synthesis of new histones during the S phase (reviewed in [20]). Therefore, the predominant way of turning over histones in G_0_ is probably transcription by RNA pol II which is linked to PAF1C activity. The PAF1 complex in budding yeast was recently shown to be structurally linked to a nucleosome disassembly step during transcription elongation [21]. In this process, nucleosome octamers are partially disassembled into smaller hexameric structures via interactions with PAF1C. The LEO1 subunit has a key position to interact with the nucleosome and facilitate histone exchange during this process. Our finding in fission yeast of a reduced turnover of histone H3 in the *ΔLEO1* mutant is consistent with this notion. Paf1C is a functionally and structurally conserved complex [12] Therefore we speculate that an akin mechanism also occurs in human G_0_ cells in which PAF1C reduces H3K9me2 levels by stimulating the turnover of histone H3.

### 4.4. A Conserved Role of LEO1 in Quiescent Eukaryotes?

We have found a role for LEO1 in the regulation of gene expression and suppression of H3K9 methylation during quiescence both in fission yeast and human cells. However, the phenotype of *LEO1* gene deleted cells is different between the two species. In human fibroblasts, LEO1 is required for efficient entry into quiescence, whereas in fission yeast LEO1 is dispensable for entry into quiescence but required for long-term survival in quiescence. This could be explained by different target genes being affected by *ΔLEO1* in the two cases. In fission yeast *LEO1* deletion causes failure to induce genes encoding membrane transporters in quiescence leading to a reduced uptake of nutrients. Human *ΔLEO1* cells fail to induce genes involved in Calcium ion transport and cell migration in low serum conditions. This could contribute to the observed abnormal cell shape and a delay in entry into G_0_.

### 4.5. Implications

Our results may be relevant to how stem cells and cancer cells enter and exit quiescence. The results also identify LEO1 as a putative drug target. It has been shown that high cellular LEO1 levels are correlated to impaired survival of lung cancer [22]. Hence, targeting LEO1 may be a new option for cancer therapy.

## 5. Conclusions

Our RNA-sequencing methodology allowed us to conclude that gene expression is generally reduced in serum-deprived quiescent human fibroblasts. Cells harboring a gene knockout for the PAF1C subunit LEO1 are viable and show a phenotype with slow entry into quiescence. LEO1 is important for proper regulation of gene expression and histone H3K9 methylation in quiescence. Thus, LEO1 is implicated in the regulation of gene expression and epigenome changes during quiescence both in fission yeast and human cells.

## Data Availability

The LEO1 knockout cell line can be requested by writing to KE. The RNA-seq and ChIP-seq data have been submitted to the NCBI Gene Expression Omnibus (GEO) under the accession number GSE247832.

## Supplementary Materials

The following supporting information can be downloaded at: https://www.mdpi.com/article/10.3390/biom13111662/s1, Figure S1: Original Western blot with loading control; Figure S2: (A) MTT assay with four replicates (B) FACS analysis in triplicates (C) Cell morphology change statistics; Figure S3: ChIP-seq library controls; Supplementary Tables S1–S4: Complete gene ontology analysis; Supplementary Table S5: RNA-seq gene lists ERCC normalized.

## Abbreviations

LEO1	Left open reading frame 1
*ΔLEO1*	gene deletion of LEO1
WT	wild type
PAF1C	RNA polymerase II associated factor 1 complex
HES1	Hairy and Enhancer of Split-1
CRISPR	Clustered regularly interspaced short palindromic repeats
H3K9	histone H3 lysine 9
BJ-5ta	hTERT-immortalized fibroblasts cell line
FBS	fetal bovine serum
ChIP	Chromatin immunoprecipitation
FACS	Fluorescence-activated cell sorting
ERCC	External RNA Controls Consortium
MTT	(3-(4,5-dimethylthiazol-2-yl)-2,5 diphenyl tetrazolium bromide)
ERK1/ERK2	Extracellular signal-regulated kinase 1/2
Wnt	Wingless and Int-1
TSS	Transcription start site
