# Supplementary material for: LEO1 Is Required for Efficient Entry into Quiescence, Control of H3K9 Methylation and Gene Expression in Human Fibroblasts
Source: Biomolecules. 2023 Nov 17;13(11):1662. doi: 10.3390/biom13111662 (PMC10668985; doi:10.3390/biom13111662)
Supplement: Supplementary file 1 [file biomolecules-13-01662-s001.zip › Supplementary Figure S1.pdf]

A)

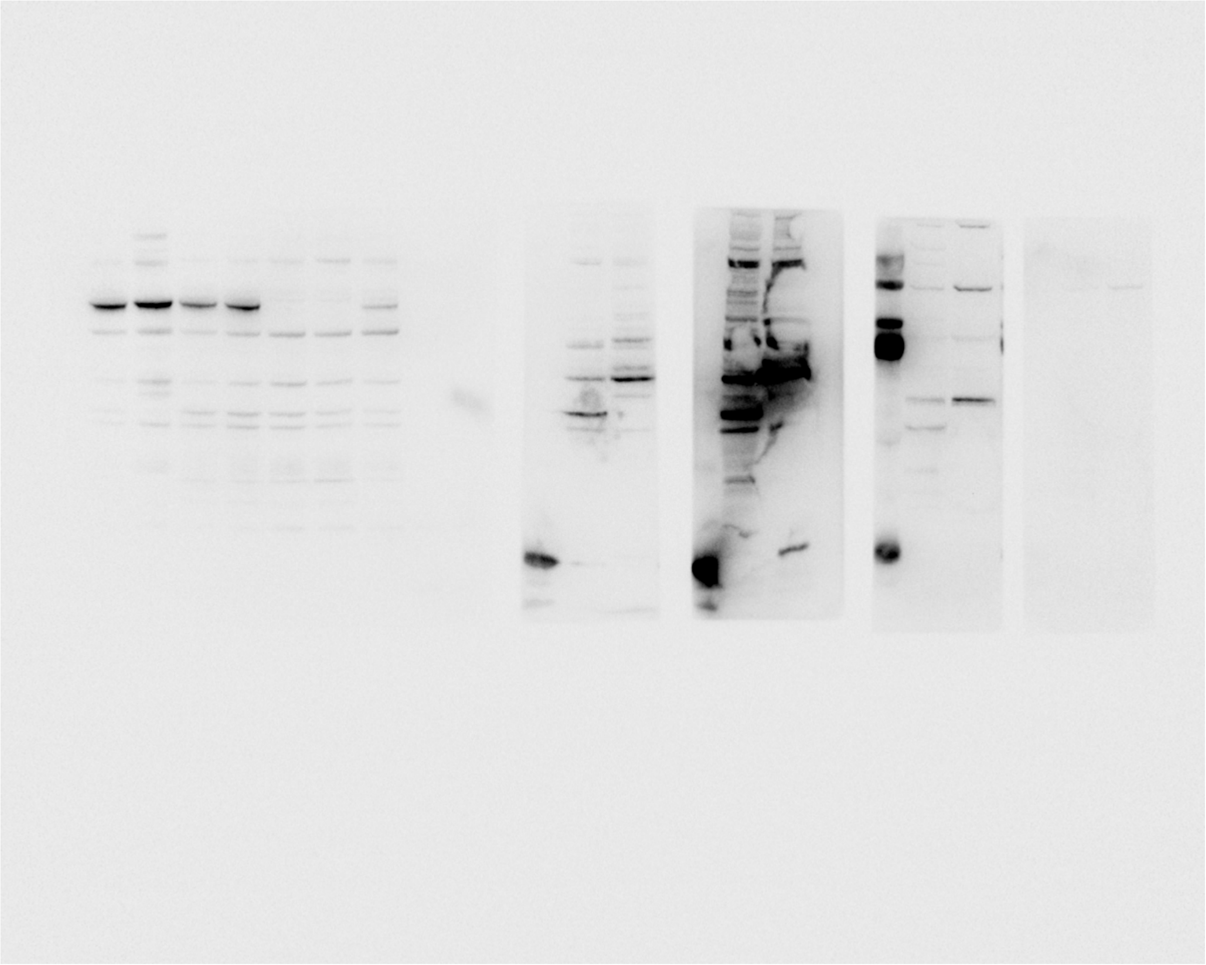

B)

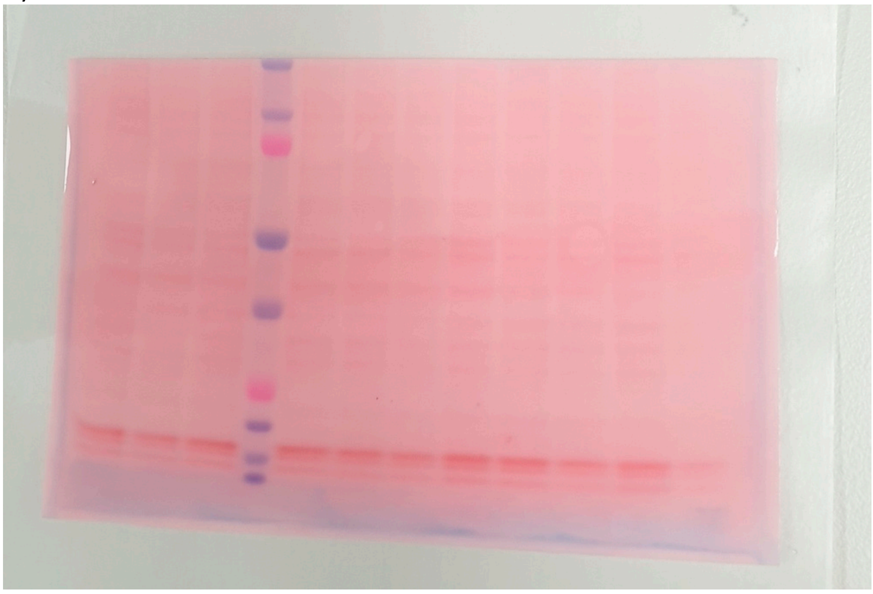

Legend: (A) The original uncropped image of Western blot in Figure 1 (left side; 7 lanes). The BJ-5ta CRISPR-Cas9 knockout clones were tested by Western blot using the anti-LEO1 A300-175A antibody (BETHYL company) at 1/1000 dilution. (B) The picture shows RED Ponceau stain of gel before cutting of gel (cut next to size marker right side; 7 lanes) and transfer for Western blot.
