# Supplementary material for: LEO1 Is Required for Efficient Entry into Quiescence, Control of H3K9 Methylation and Gene Expression in Human Fibroblasts
Source: Biomolecules. 2023 Nov 17;13(11):1662. doi: 10.3390/biom13111662 (PMC10668985; doi:10.3390/biom13111662)

A) MTT assay with four replicates

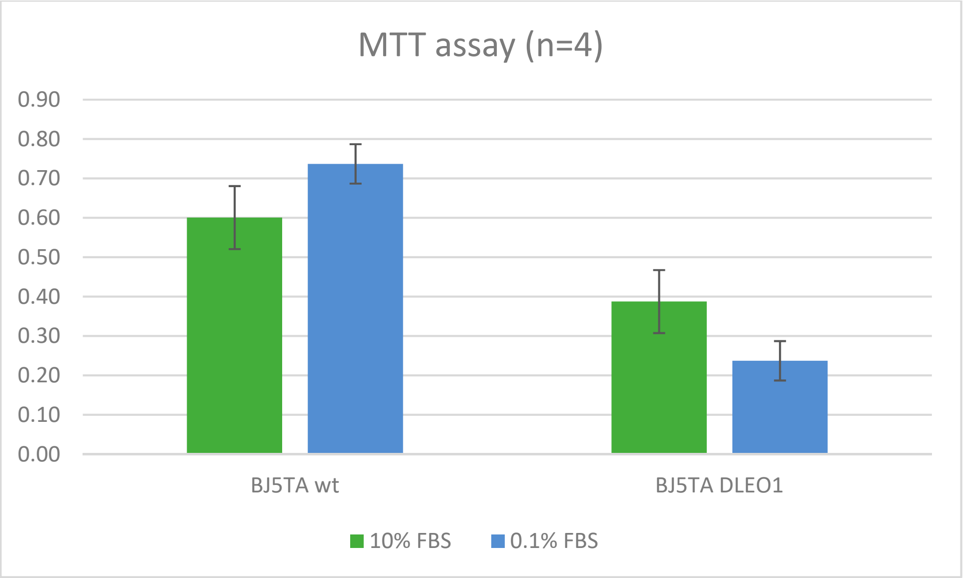

B) FACS analysis in triplicates

T-0
WT

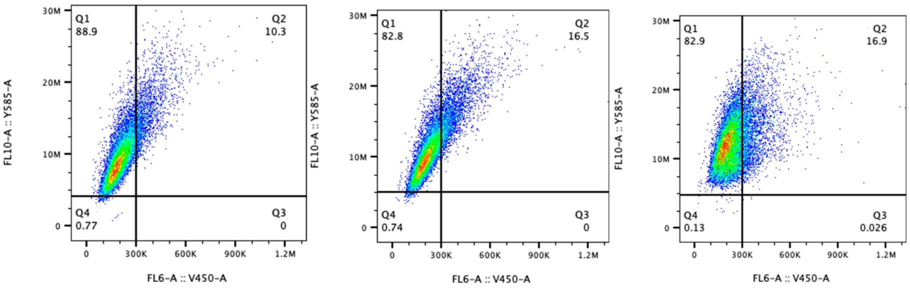

T-0
Leo1

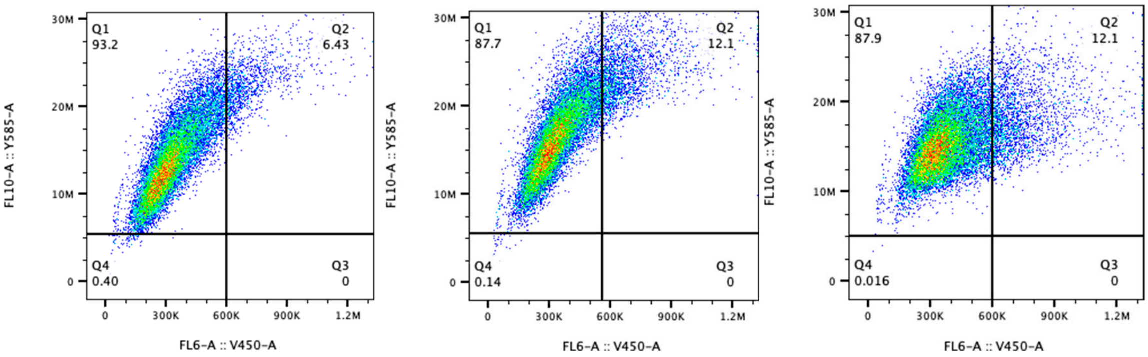

T-1D
WT

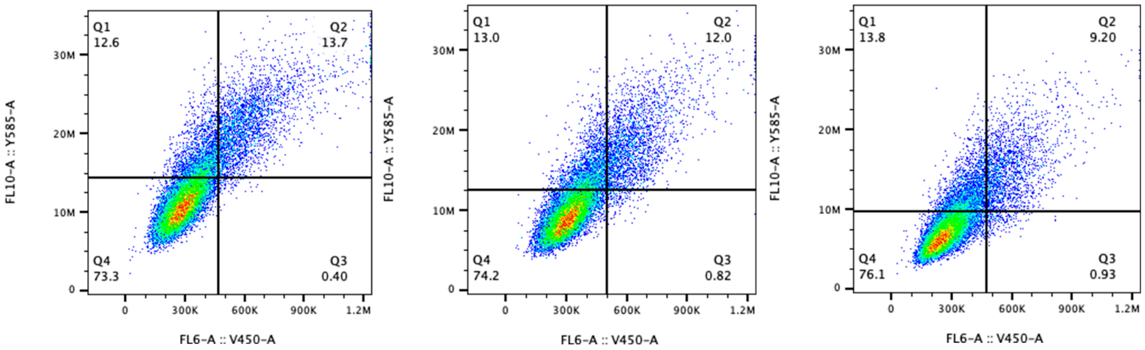

T1-D
Leo1

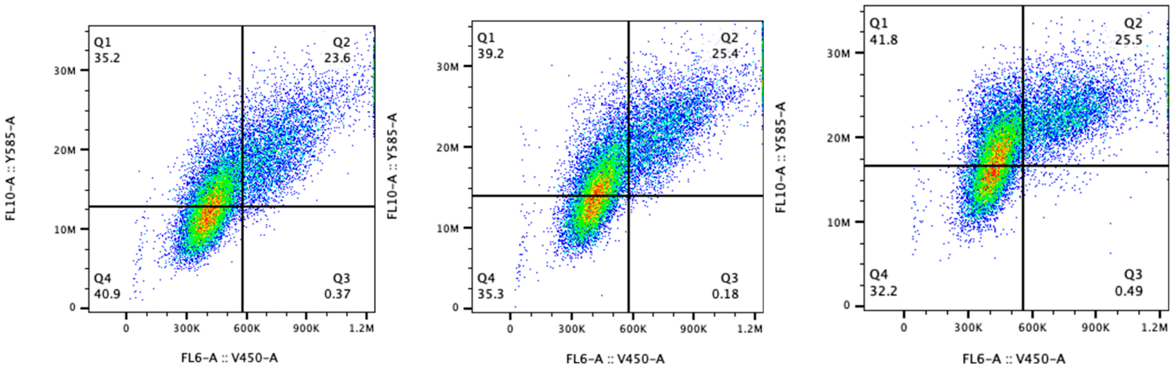

T-3D
WT

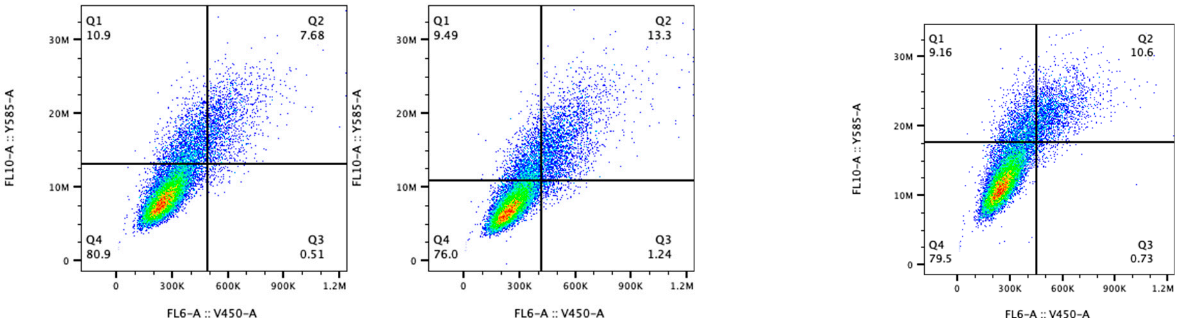

T3-D
Leo1

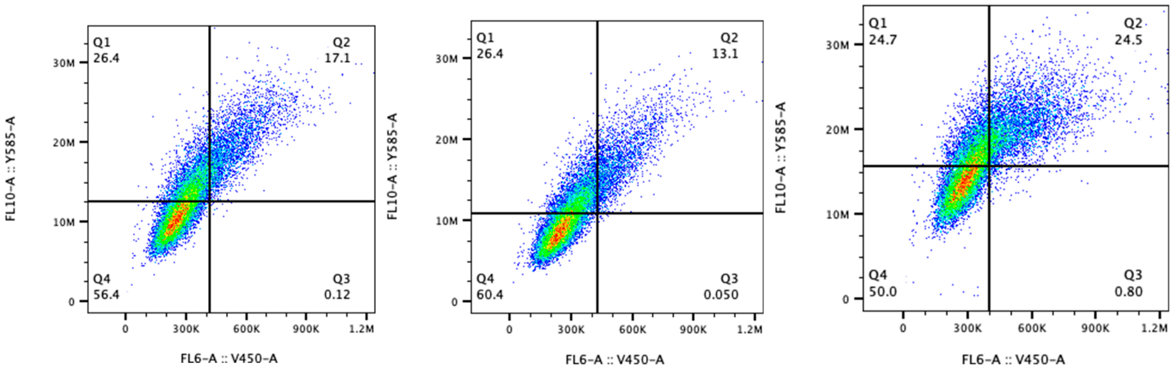

T-6D

WT

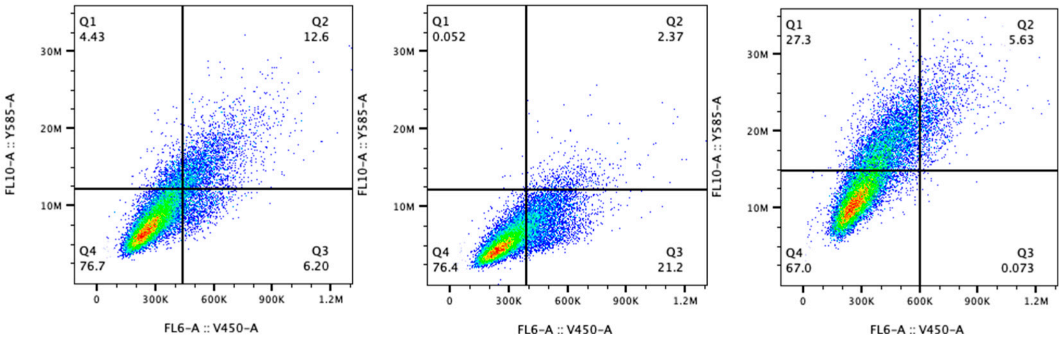

T6-D Leo1

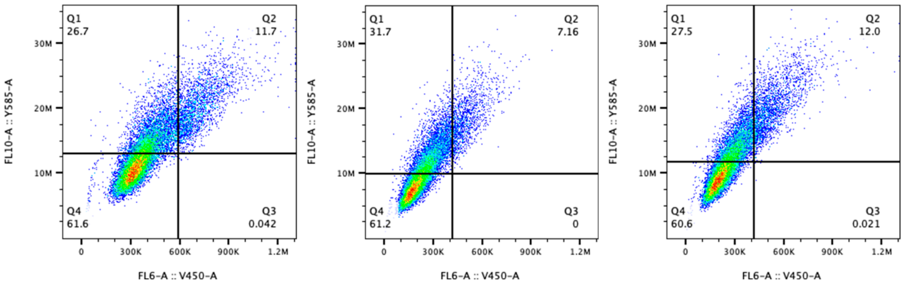

T-9D

WT

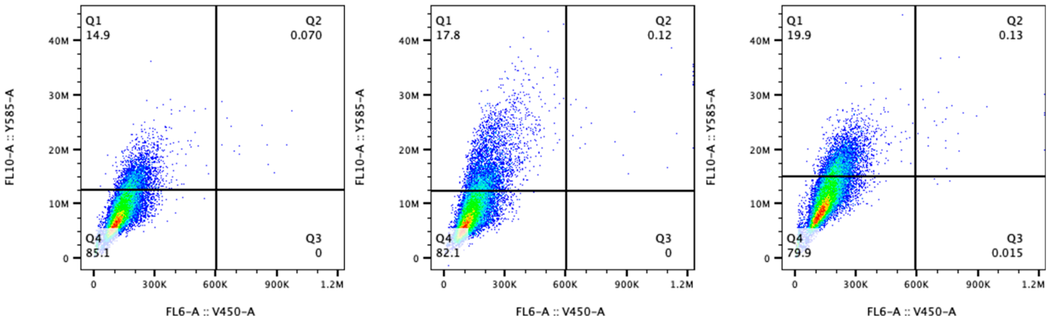

T9-D Leo1

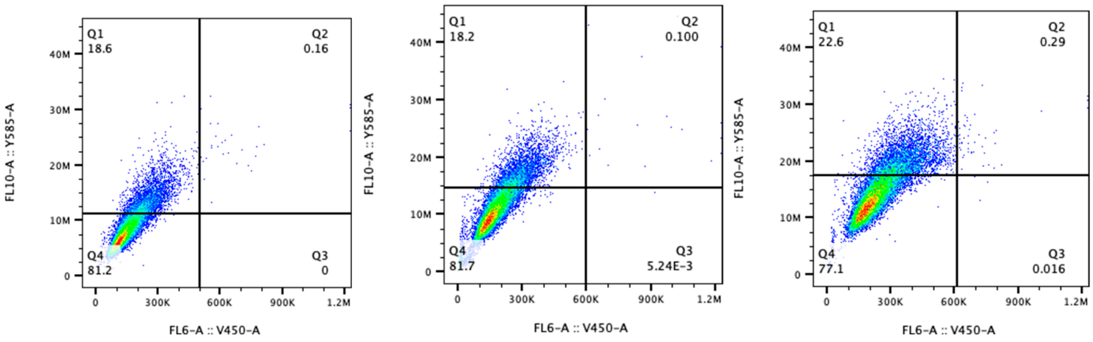

C) Cell morphology change statistics

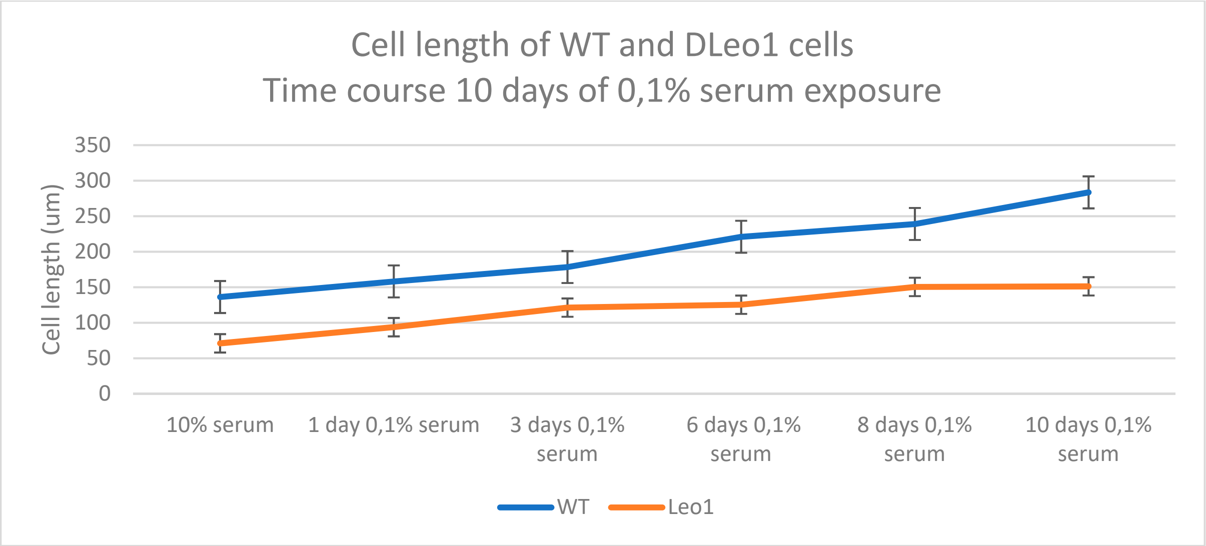

Supplement: Supplementary file 1 [file biomolecules-13-01662-s001.zip › Supplementary Figure S2.pdf]
