# Supplementary material for: LEO1 Is Required for Efficient Entry into Quiescence, Control of H3K9 Methylation and Gene Expression in Human Fibroblasts
Source: Biomolecules. 2023 Nov 17;13(11):1662. doi: 10.3390/biom13111662 (PMC10668985; doi:10.3390/biom13111662)
Supplement: Supplementary file 1 [file biomolecules-13-01662-s001.zip › Supplementary Figure S3.pdf]

Filename: BEA20P105_LC library QC 2020-08-17-01.HSD1000

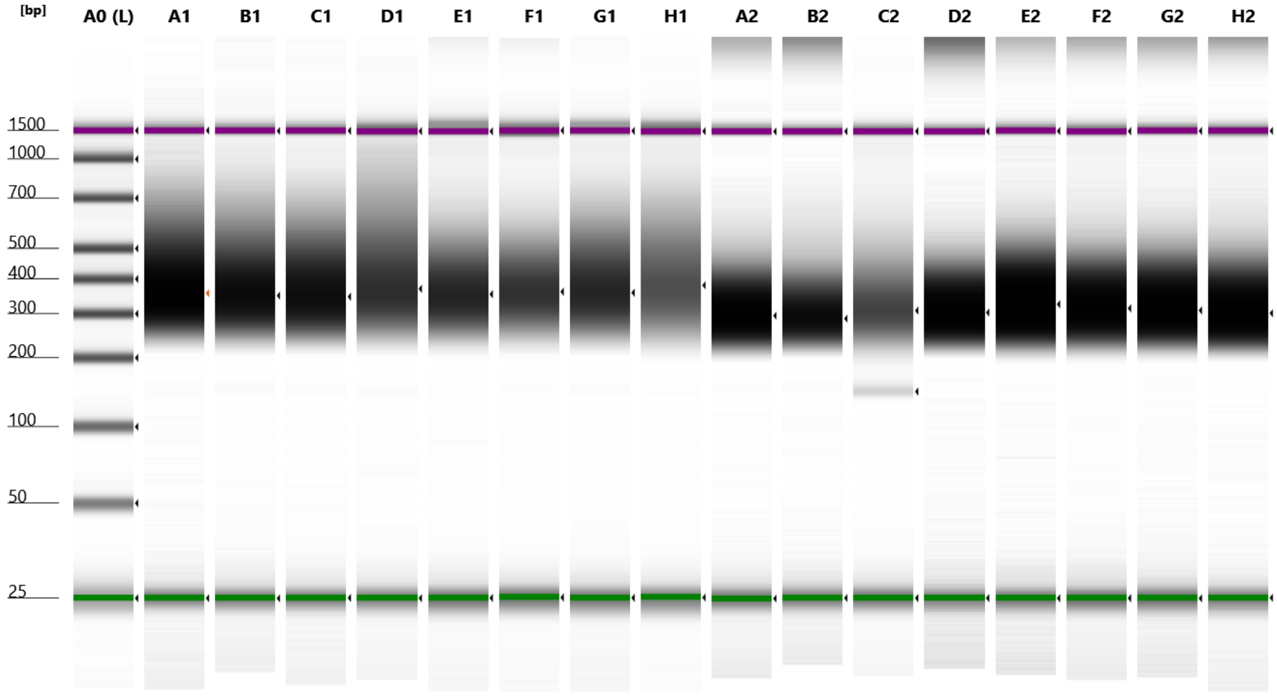

Default image (Contrast 100%)

Sample Info

Well	Conc. [pg/µl]	Sample Description	Alert	Observations
A0	2350	Electronic Ladder		Ladder
A1	5120	BEA20P105_LC1_H3K9Me2		
B1	4090	BEA20P105_LC2_H3K9Me2		
C1	4320	BEA20P105_LC3_H3K9Me2		
D1	2340	BEA20P105_LC4_H3K9Me2		
E1	2470	BEA20P105_LC5_H3K9Me2		
F1	1870	BEA20P105_LC6_H3K9Me2		
G1	2810	BEA20P105_LC7_H3K9Me2		
H1	1120	BEA20P105_LC8_H3K9Me2		
A2	4890	BEA20P105_LC9_Input1		
B2	3720	BEA20P105_LC10_Input2		
C2	2160	BEA20P105_LC11_Input3 (non-diluted)		
D2	5560	BEA20P105_LC12_Input4		
E2	6850	BEA20P105_LC13_Input5		
F2	4770	BEA20P105_LC14_Input6		
G2	5610	BEA20P105_LC15_Input7		
H2	4980	BEA20P105_LC16_Input8		

© Agilent Technologies, Inc. 2021

A0: Electronic Ladder

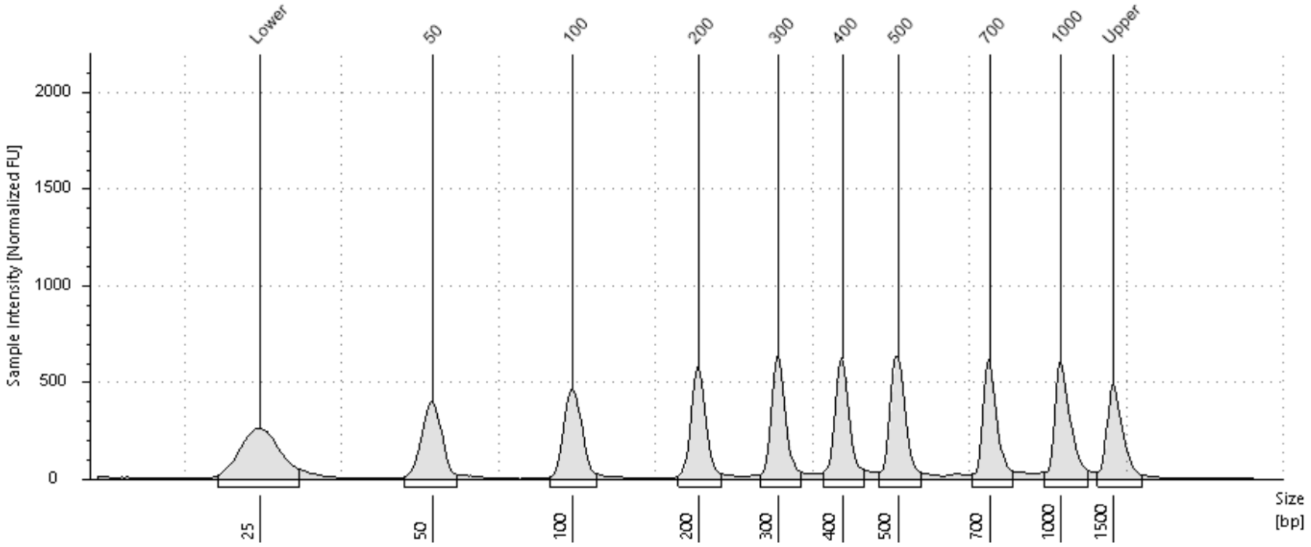

Sample Table

Well	Conc. [pg/µl]	Sample Description	Alert	Observations
A0	2350	Electronic Ladder		Ladder

Peak Table

Size [bp]	Calibrated Conc. [pg/µl]	Assigned Conc. [pg/µl]	Peak Molarity [pmol/l]	% Integrated Area	Peak Comment	Observations
25	340	-	20900	-		Lower Marker
50	265	-	8160	11.28		
100	278	-	4270	11.82		
200	290	-	2230	12.32		
300	304	-	1560	12.95		
400	306	-	1180	13.00		
500	312	-	961	13.29		
700	286	-	629	12.19		
1000	309	-	476	13.15		
1500	250	250	256	-		Upper Marker

© Agilent Technologies, Inc. 2021

A1: BEA20P105_LC1_H3K9Me2

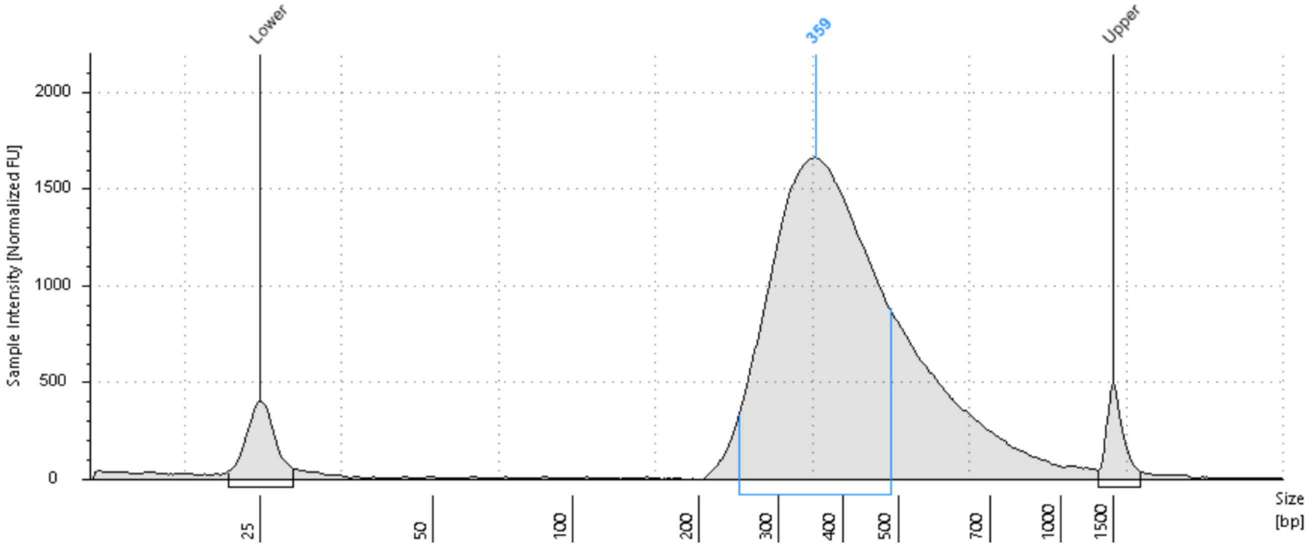

Sample Table

Well	Conc. [pg/µl]	Sample Description	Alert	Observations
A1	5120	BEA20P105_LC1_H3K9Me2		

Peak Table

Size [bp]	Calibrated Conc. [pg/µl]	Assigned Conc. [pg/µl]	Peak Molarity [pmol/l]	% Integrated Area	Peak Comment	Observations
25	367	-	22600	-		Lower Marker
359	5120	-	21900	100.00		
1500	250	250	256	-		Upper Marker

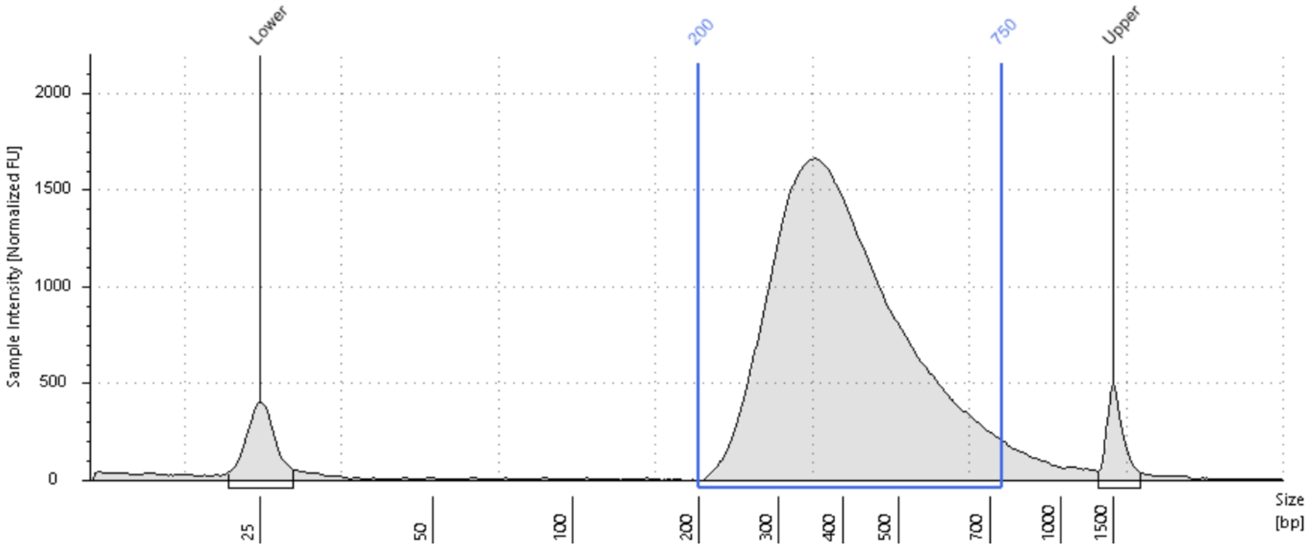

Region Table

From [bp]	To [bp]	Average Size [bp]	Conc. [pg/µl]	Region Molarity [pmol/l]	% of Total	Region Comment	Color
200	750	412	6710	26900	94.14		

© Agilent Technologies, Inc. 2021

B1: BEA20P105_LC2_H3K9Me2

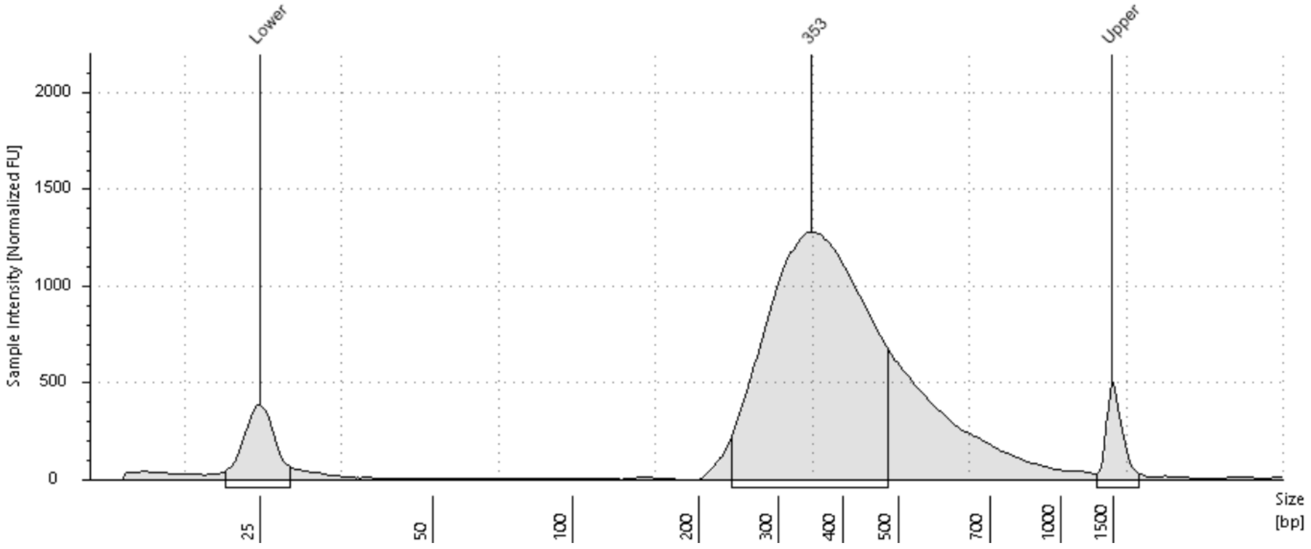

Sample Table

Well	Conc. [pg/µl]	Sample Description	Alert	Observations
B1	4090	BEA20P105_LC2_H3K9Me2		

Peak Table

Size [bp]	Calibrated Conc. [pg/µl]	Assigned Conc. [pg/µl]	Peak Molarity [pmol/l]	% Integrated Area	Peak Comment	Observations
25	381	-	23500	-		Lower Marker
353	4090	-	17800	100.00		
1500	250	250	256	-		Upper Marker

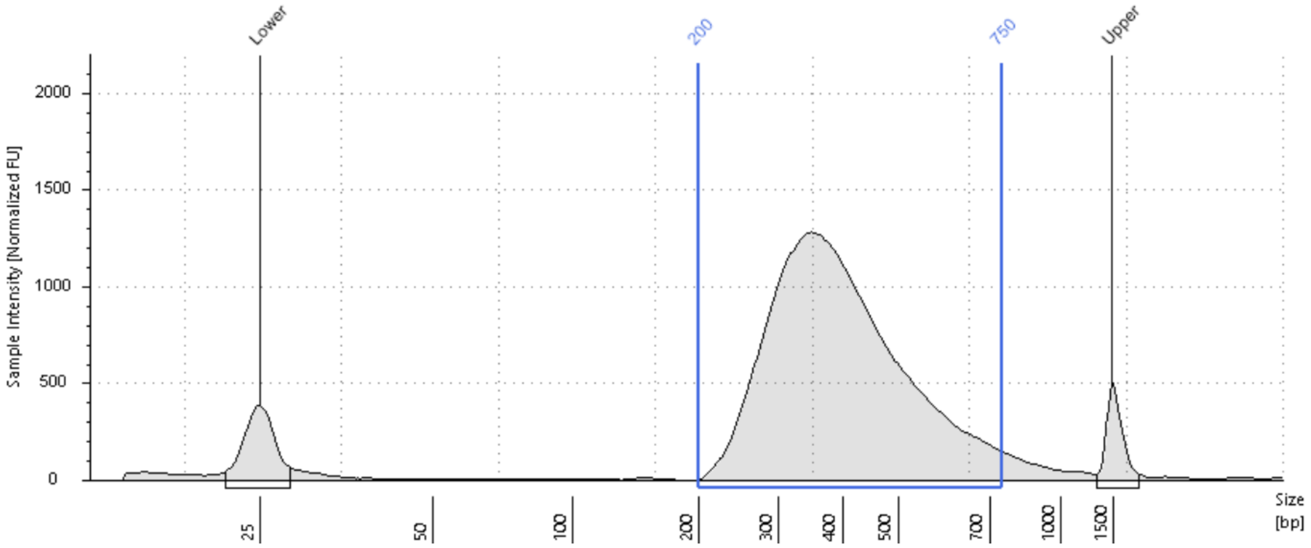

Region Table

From [bp]	To [bp]	Average Size [bp]	Conc. [pg/µl]	Region Molarity [pmol/l]	% of Total	Region Comment	Color
200	750	406	5280	21400	93.67		

© Agilent Technologies, Inc. 2021

C1: BEA20P105_LC3_H3K9Me2

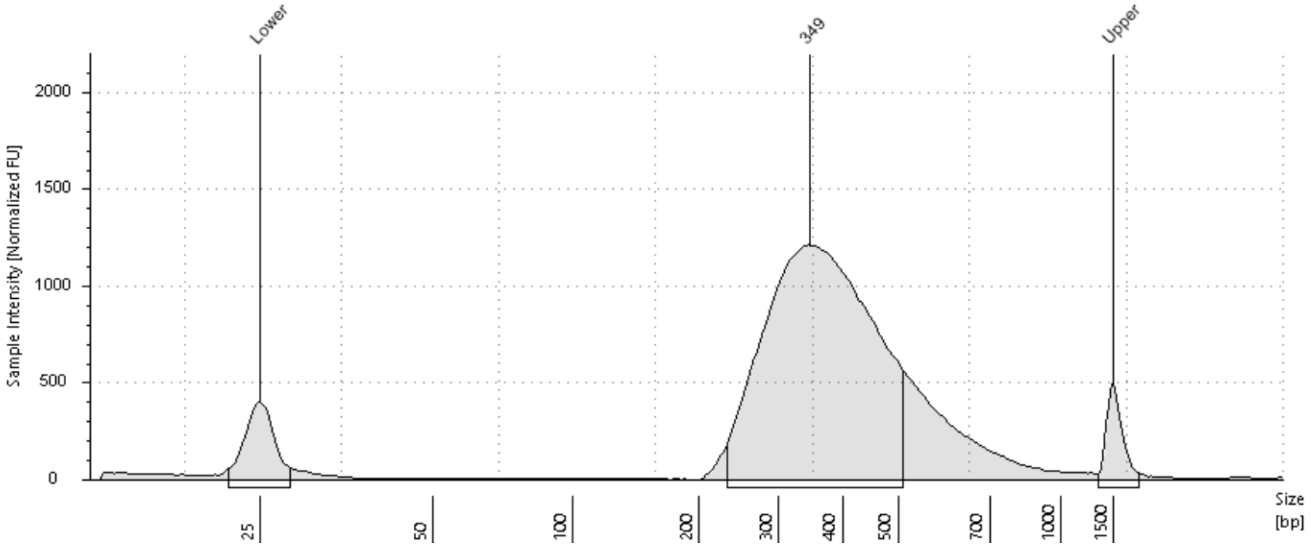

Sample Table

Well	Conc. [pg/µl]	Sample Description	Alert	Observations
C1	4320	BEA20P105_LC3_H3K9Me2		

Peak Table

Size [bp]	Calibrated Conc. [pg/µl]	Assigned Conc. [pg/µl]	Peak Molarity [pmol/l]	% Integrated Area	Peak Comment	Observations
25	379	-	23400	-		Lower Marker
349	4320	-	19000	100.00		
1500	250	250	256	-		Upper Marker

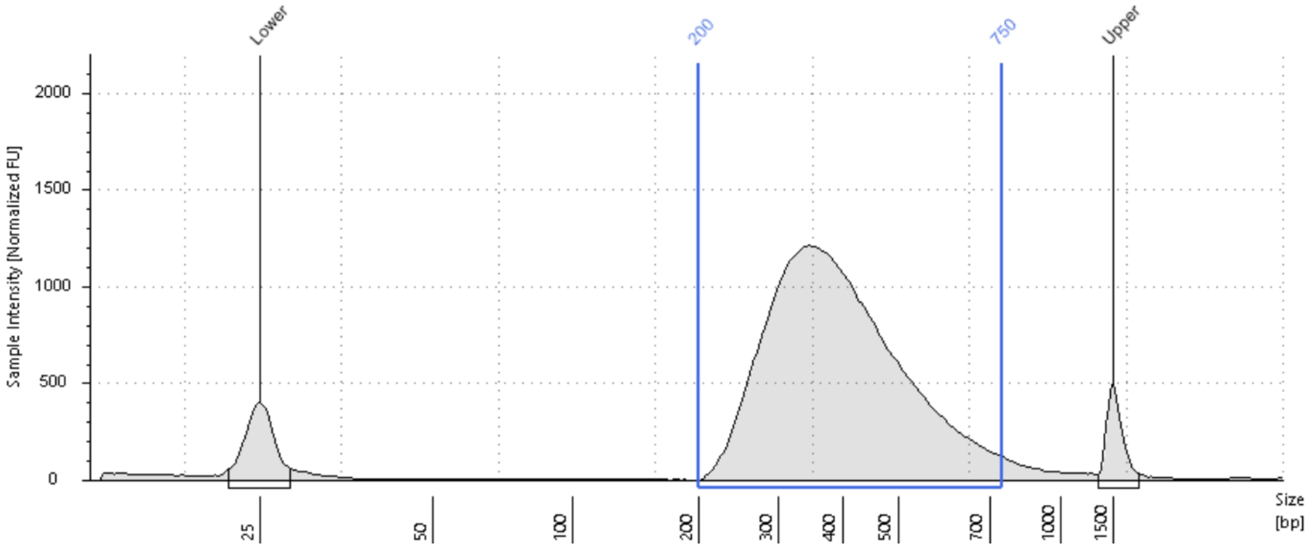

Region Table

From [bp]	To [bp]	Average Size [bp]	Conc. [pg/µl]	Region Molarity [pmol/l]	% of Total	Region Comment	Color
200	750	403	5200	21300	94.64		

© Agilent Technologies, Inc. 2021

D1: BEA20P105_LC4_H3K9Me2

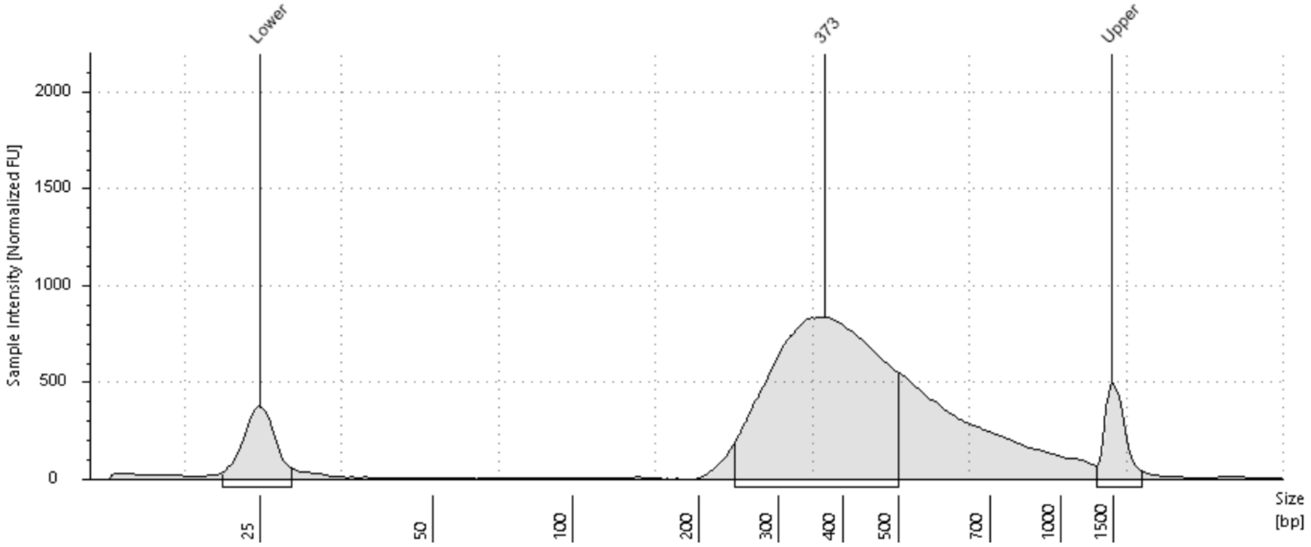

Sample Table

Well	Conc. [pg/µl]	Sample Description	Alert	Observations
D1	2340	BEA20P105_LC4_H3K9Me2		

Peak Table

Size [bp]	Calibrated Conc. [pg/µl]	Assigned Conc. [pg/µl]	Peak Molarity [pmol/l]	% Integrated Area	Peak Comment	Observations
25	305	-	18800	-		Lower Marker
373	2340	-	9650	100.00		
1500	250	250	256	-		Upper Marker

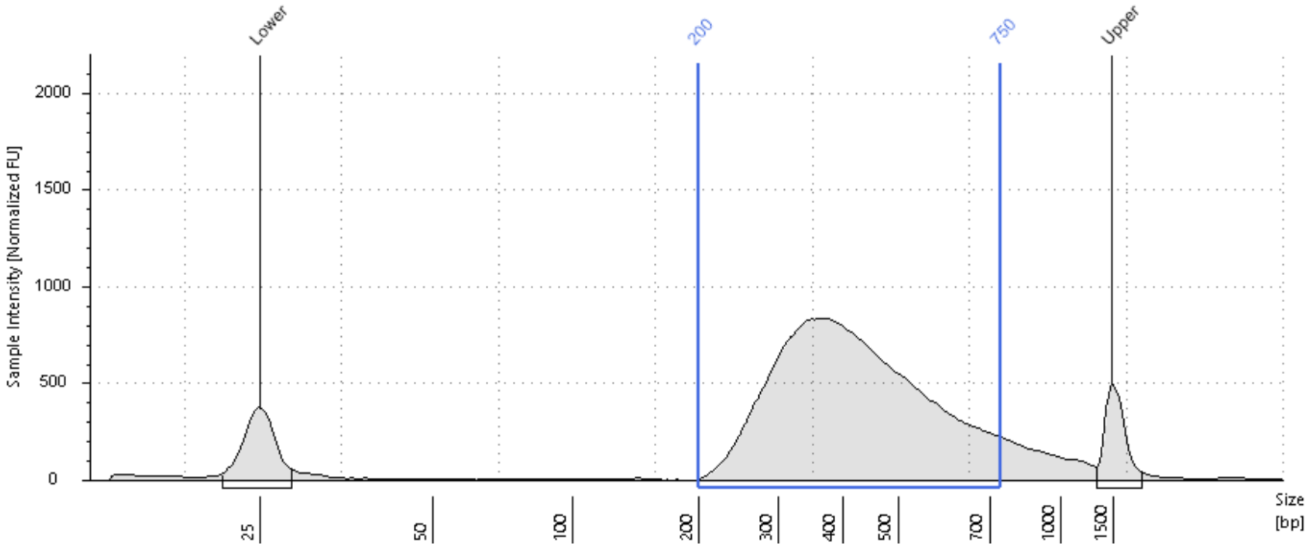

Region Table

From [bp]	To [bp]	Average Size [bp]	Conc. [pg/µl]	Region Molarity [pmol/l]	% of Total	Region Comment	Color
200	750	429	3210	12500	89.26		

© Agilent Technologies, Inc. 2021

E1: BEA20P105_LC5_H3K9Me2

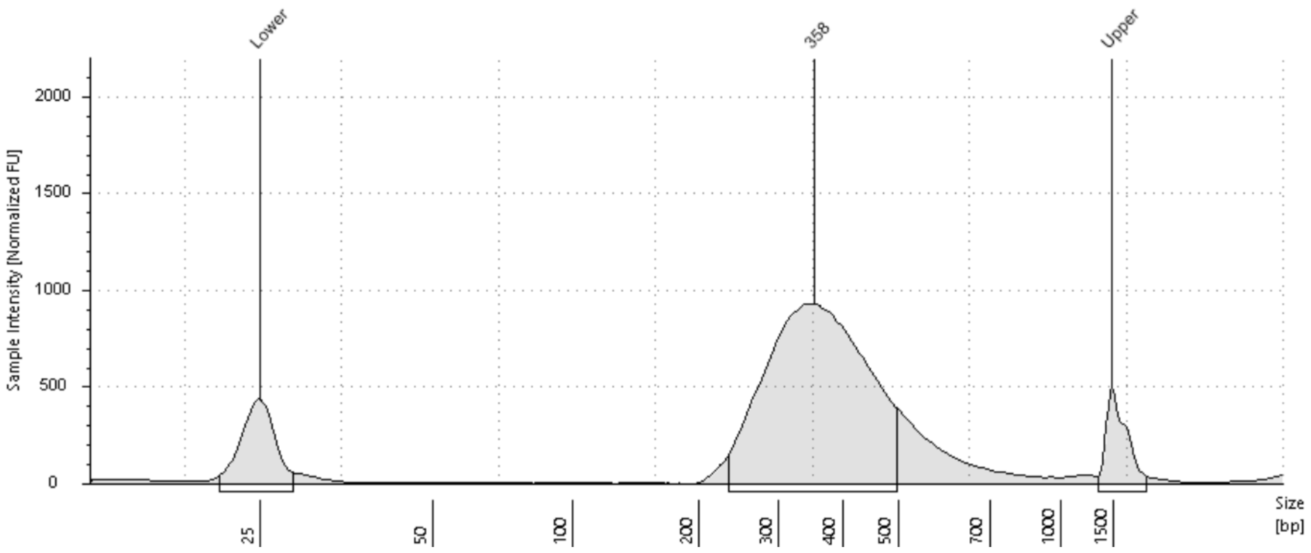

Sample Table

Well	Conc. [pg/µl]	Sample Description	Alert	Observations
E1	2470	BEA20P105_LC5_H3K9Me2		

Peak Table

Size [bp]	Calibrated Conc. [pg/µl]	Assigned Conc. [pg/µl]	Peak Molarity [pmol/l]	% Integrated Area	Peak Comment	Observations
25	375	-	23100	-		Lower Marker
358	2470	-	10600	100.00		
1500	250	250	256	-		Upper Marker

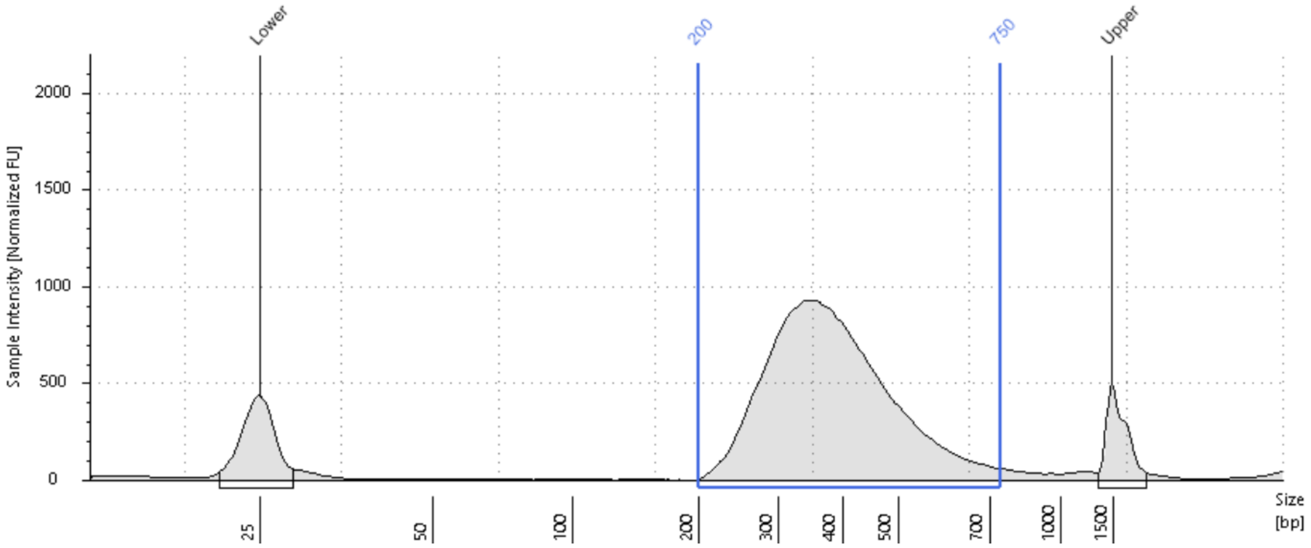

Region Table

From [bp]	To [bp]	Average Size [bp]	Conc. [pg/µl]	Region Molarity [pmol/l]	% of Total	Region Comment	Color
200	750	393	2900	12100	93.56		

© Agilent Technologies, Inc. 2021

F1: BEA20P105_LC6_H3K9Me2

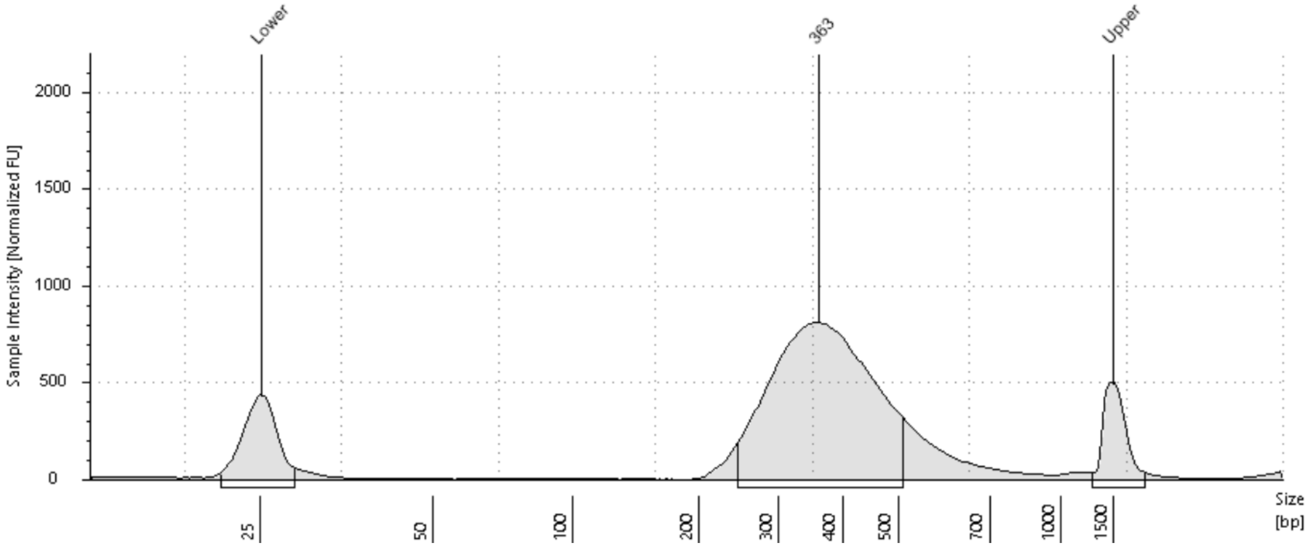

Sample Table

Well	Conc. [pg/µl]	Sample Description	Alert	Observations
F1	1870	BEA20P105_LC6_H3K9Me2		

Peak Table

Size [bp]	Calibrated Conc. [pg/µl]	Assigned Conc. [pg/µl]	Peak Molarity [pmol/l]	% Integrated Area	Peak Comment	Observations
25	334	-	20600	-		Lower Marker
363	1870	-	7920	100.00		
1500	250	250	256	-		Upper Marker

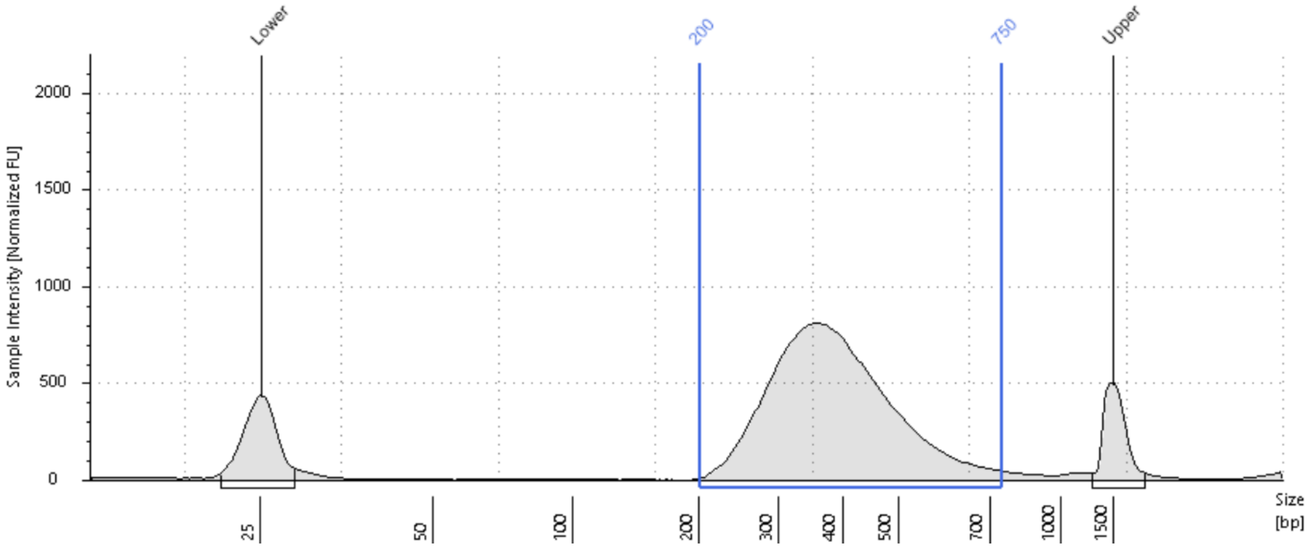

Region Table

From [bp]	To [bp]	Average Size [bp]	Conc. [pg/µl]	Region Molarity [pmol/l]	% of Total	Region Comment	Color
200	750	394	2180	9070	94.37		

© Agilent Technologies, Inc. 2021

G1: BEA20P105_LC7_H3K9Me2

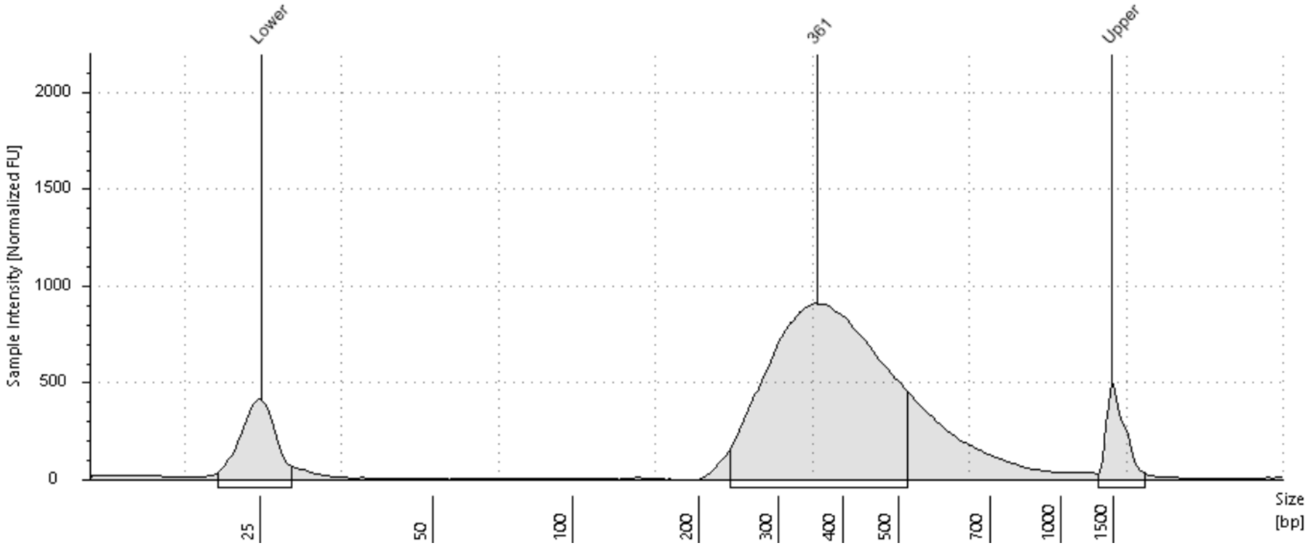

Sample Table

Well	Conc. [pg/µl]	Sample Description	Alert	Observations
Gl	2810	BEA20P105_LC7_H3K9Me2		

Peak Table

Size [bp]	Calibrated Conc. [pg/µl]	Assigned Conc. [pg/µl]	Peak Molarity [pmol/l]	% Integrated Area	Peak Comment	Observations
25	399	-	24600	-		Lower Marker
361	2810	-	12000	100.00		
1500	250	250	256	-		Upper Marker

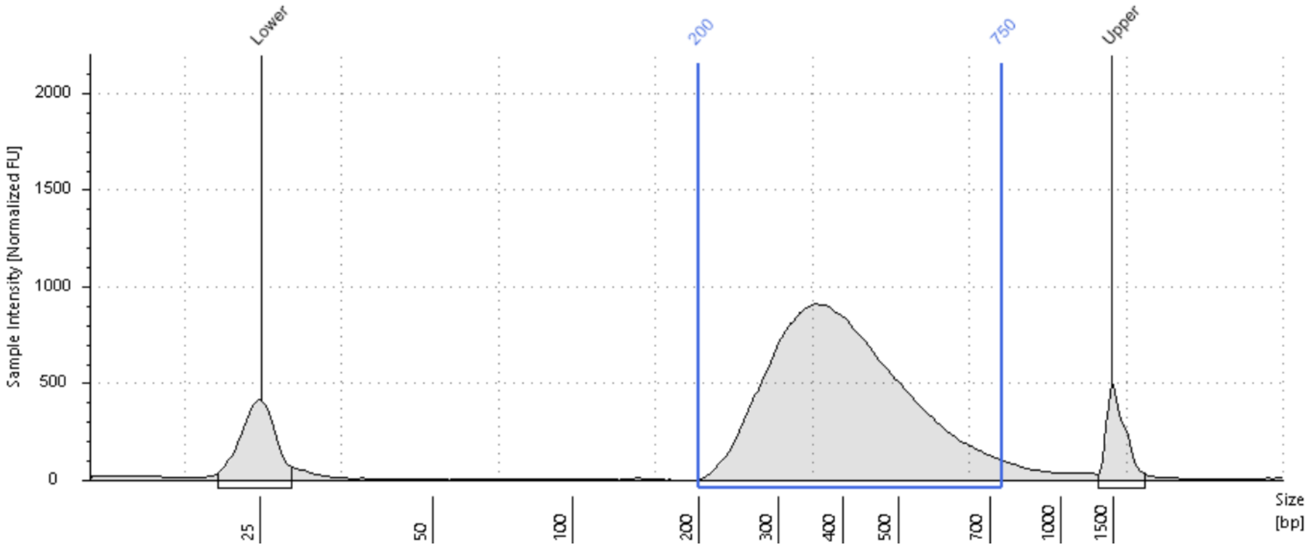

Region Table

From [bp]	To [bp]	Average Size [bp]	Conc. [pg/µl]	Region Molarity [pmol/l]	% of Total	Region Comment	Color
200	750	410	3410	13700	93.83		

© Agilent Technologies, Inc. 2021

H1: BEA20P105_LC8_H3K9Me2

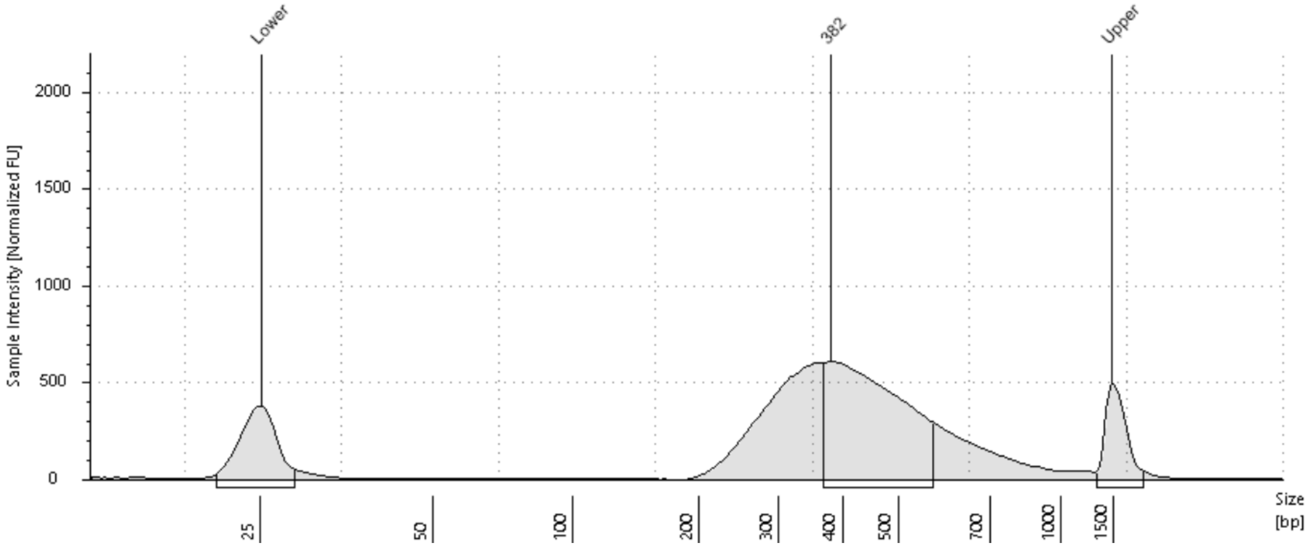

Sample Table

Well	Conc. [pg/µl]	Sample Description	Alert	Observations
H1	1120	BEA20P105_LC8_H3K9Me2		

Peak Table

Size [bp]	Calibrated Conc. [pg/µl]	Assigned Conc. [pg/µl]	Peak Molarity [pmol/l]	% Integrated Area	Peak Comment	Observations
25	338	-	20800	-		Lower Marker
382	1120	-	4510	100.00		
1500	250	250	256	-		Upper Marker

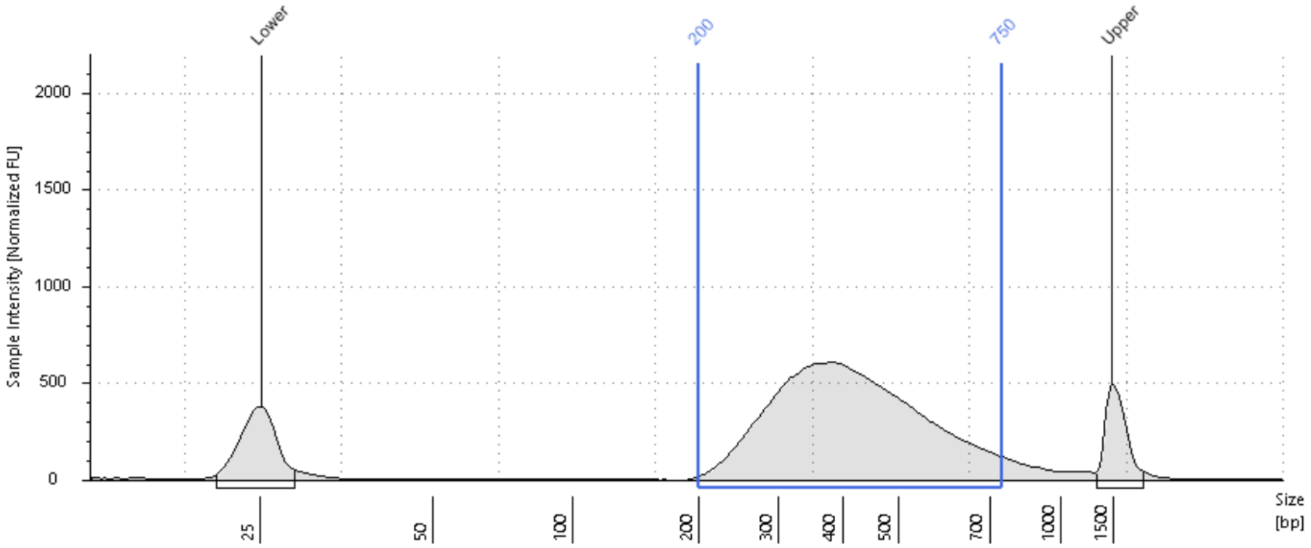

Region Table

From [bp]	To [bp]	Average Size [bp]	Conc. [pg/µl]	Region Molarity [pmol/l]	% of Total	Region Comment	Color
200	750	424	2250	8860	92.36		

A2: BEA20P105_LC9_Input1

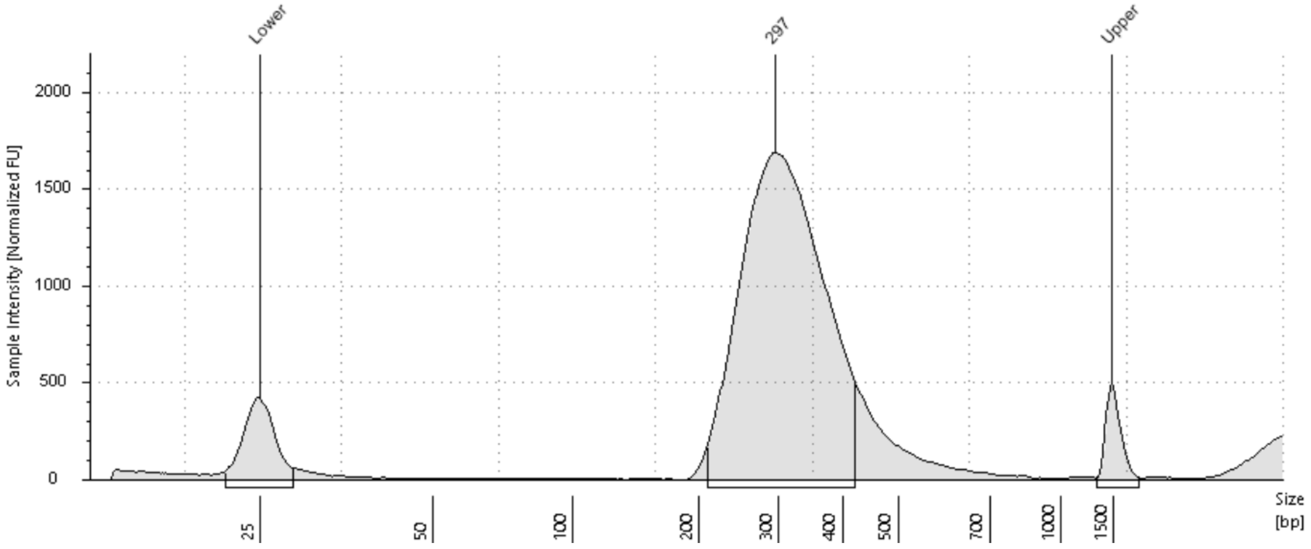

Sample Table

Well	Conc. [pg/µl]	Sample Description	Alert	Observations
A2	4890	BEA20P105_LC9_Input1		

Peak Table

Size [bp]	Calibrated Conc. [pg/µl]	Assigned Conc. [pg/µl]	Peak Molarity [pmol/l]	% Integrated Area	Peak Comment	Observations
25	456	-	28100	-		Lower Marker
297	4890	-	25300	100.00		
1500	250	250	256	-		Upper Marker

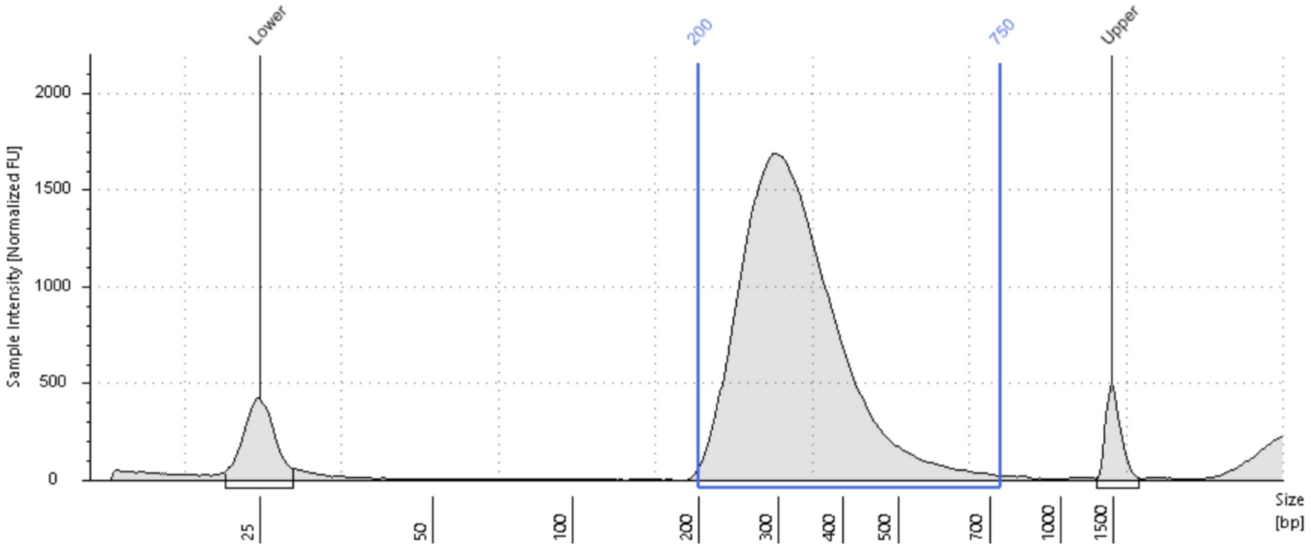

Region Table

From [bp]	To [bp]	Average Size [bp]	Conc. [pg/µl]	Region Molarity [pmol/l]	% of Total	Region Comment	Color
200	750	334	5520	26800	91.63		

© Agilent Technologies, Inc. 2021

B2: BEA20P105_LC10_Input2

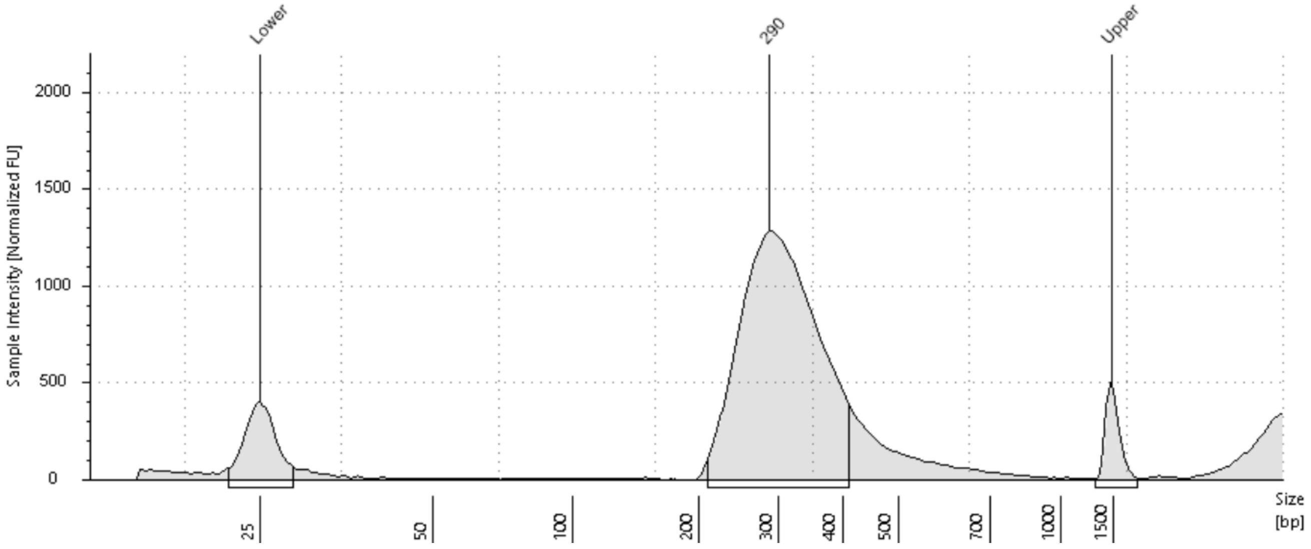

Sample Table

Well	Conc. [pg/µl]	Sample Description	Alert	Observations
B2	3720	BEA20P105_LC10_Input2		

Peak Table

Size [bp]	Calibrated Conc. [pg/µl]	Assigned Conc. [pg/µl]	Peak Molarity [pmol/l]	% Integrated Area	Peak Comment	Observations
25	460	-	28300	-		Lower Marker
290	3720	-	19800	100.00		
1500	250	250	256	-		Upper Marker

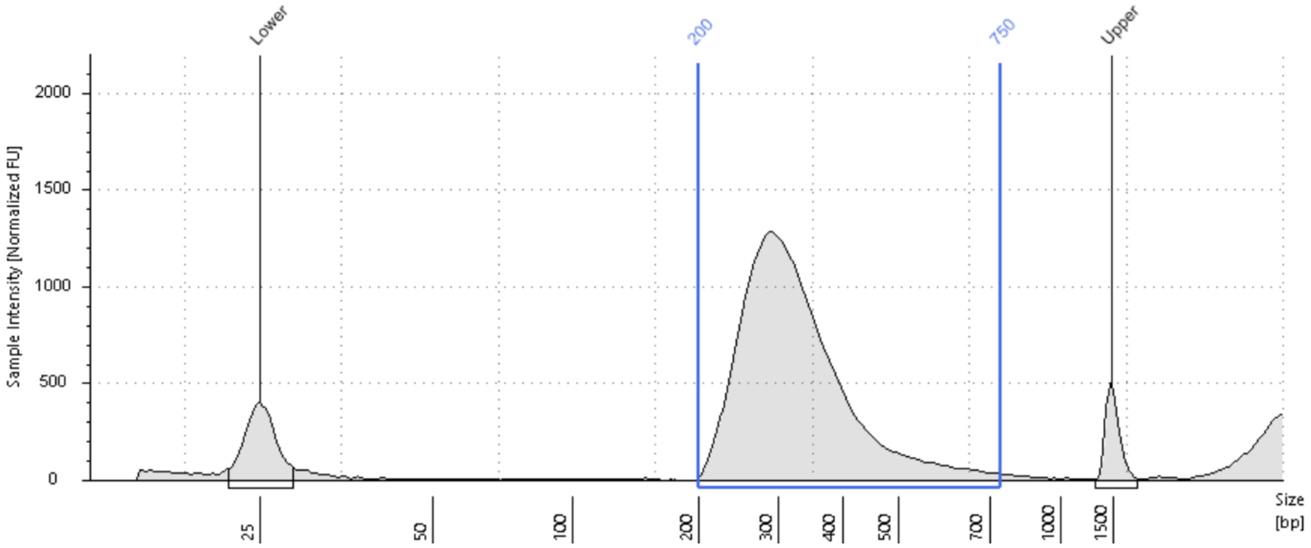

Region Table

From [bp]	To [bp]	Average Size [bp]	Conc. [pg/µl]	Region Molarity [pmol/l]	% of Total	Region Comment	Color
200	750	336	4320	21000	85.59		

© Agilent Technologies, Inc. 2021

C2: BEA20P105_LC11_Input3 (non-diluted)

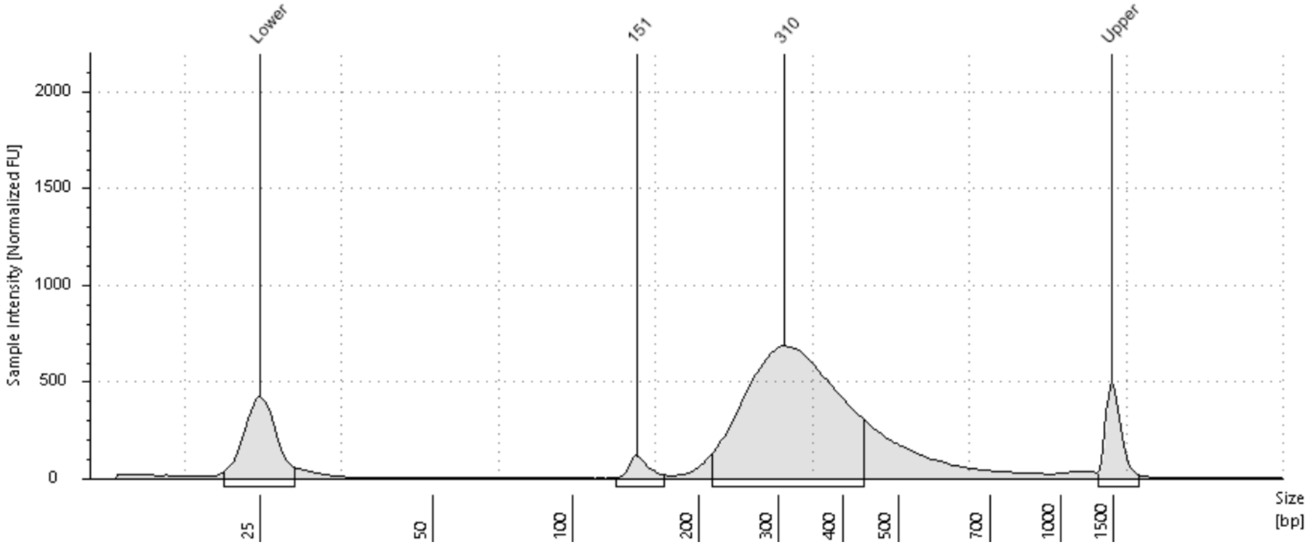

Sample Table

Well	Conc. [pg/µl]	Sample Description	Alert	Observations
C2	2160	BEA20P105_LC11_Input3 (non-diluted)		

Peak Table

Size [bp]	Calibrated Conc. [pg/µl]	Assigned Conc. [pg/µl]	Peak Molarity [pmol/l]	% Integrated Area	Peak Comment	Observations
25	447	-	27500	-		Lower Marker
151	75.0	-	762	3.47		
310	2090	-	10400	96.53		
1500	250	250	256	-		Upper Marker

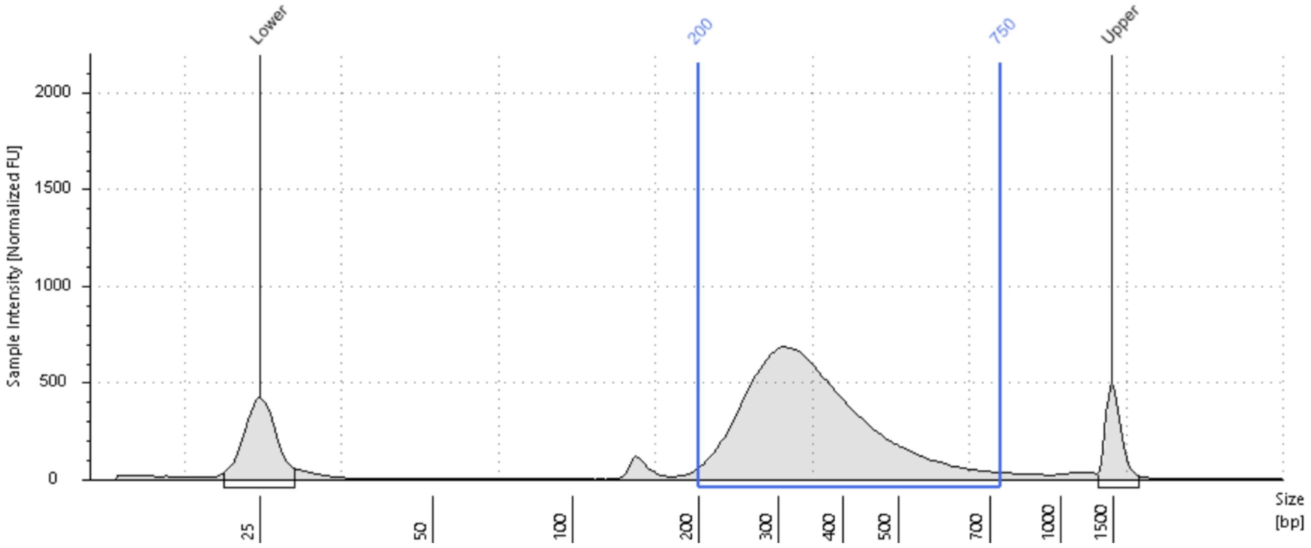

Region Table

From [bp]	To [bp]	Average Size [bp]	Conc. [pg/µl]	Region Molarity [pmol/l]	% of Total	Region Comment	Color
200	750	361	2590	11900	90.77		

© Agilent Technologies, Inc. 2021

D2: BEA20P105_LC12_Input4

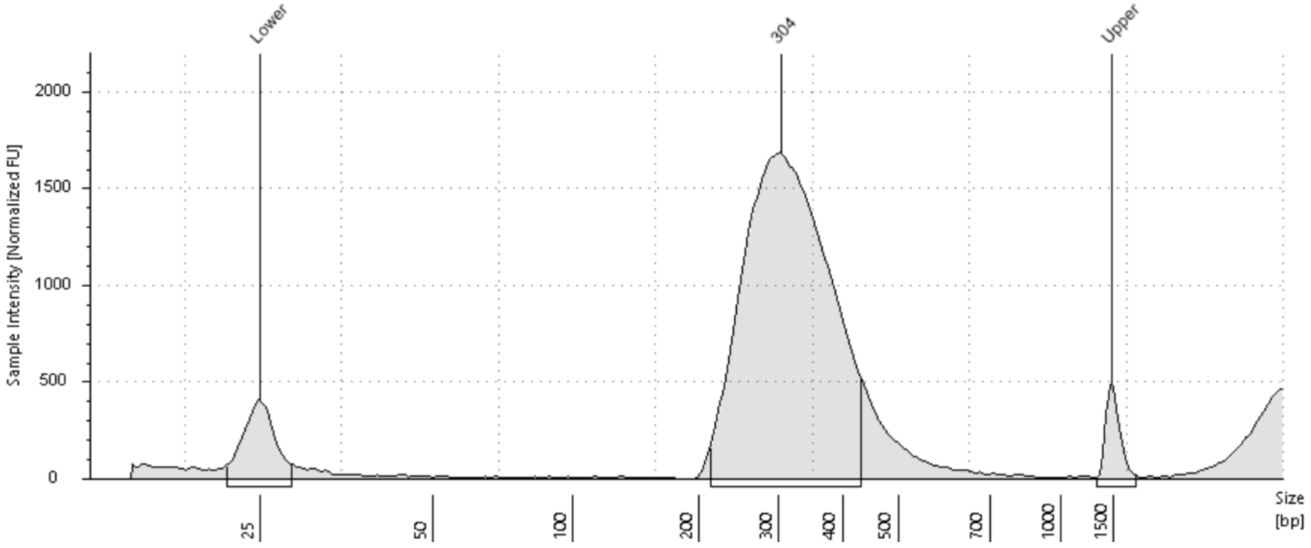

Sample Table

Well	Conc. [pg/µl]	Sample Description	Alert	Observations
D2	5560	BEA20P105_LC12_Input4		

Peak Table

Size [bp]	Calibrated Conc. [pg/µl]	Assigned Conc. [pg/µl]	Peak Molarity [pmol/l]	% Integrated Area	Peak Comment	Observations
25	476	-	29300	-		Lower Marker
304	5560	-	28100	100.00		
1500	250	250	256	-		Upper Marker

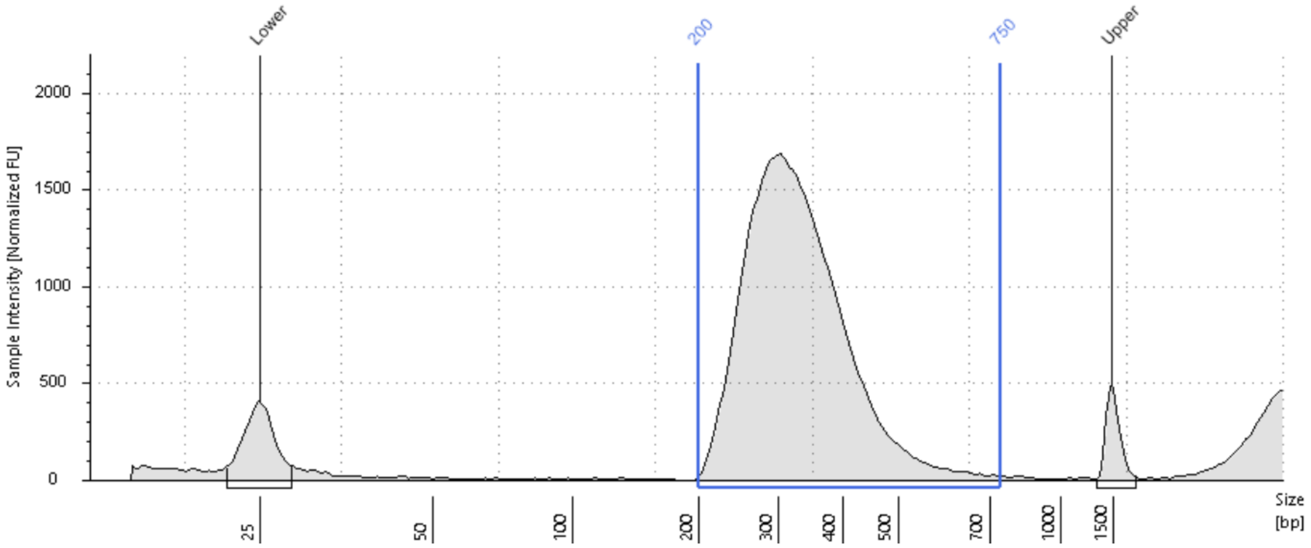

Region Table

From [bp]	To [bp]	Average Size [bp]	Conc. [pg/µl]	Region Molarity [pmol/l]	% of Total	Region Comment	Color
200	750	337	6180	29700	85.22		

© Agilent Technologies, Inc. 2021

E2: BEA20P105_LC13_Input5

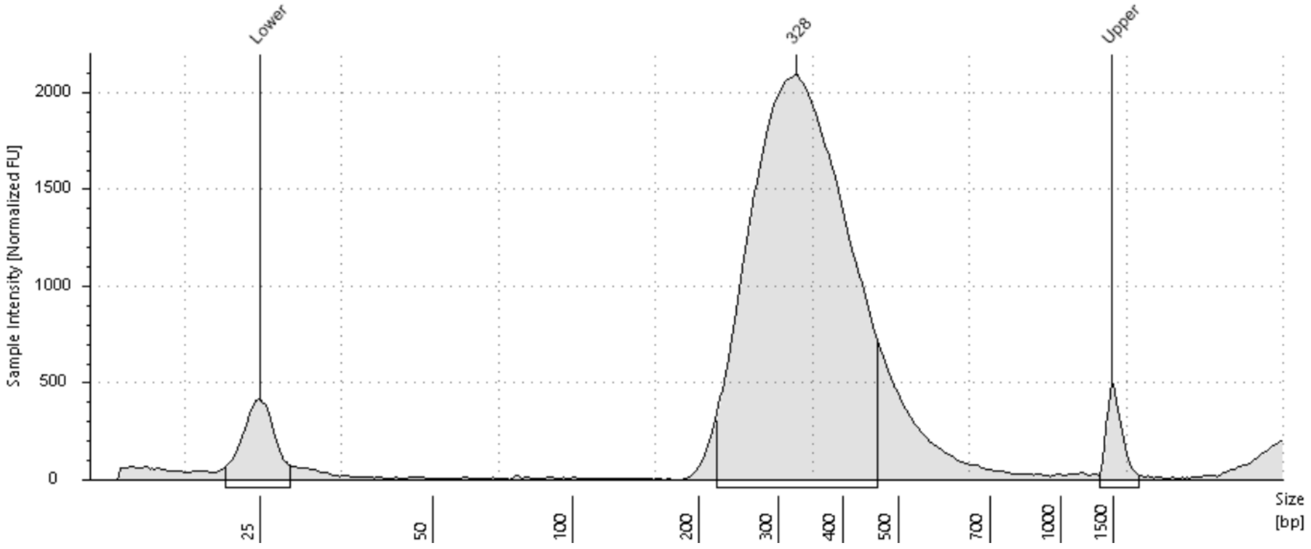

Sample Table

Well	Conc. [pg/µl]	Sample Description	Alert	Observations
E2	6850	BEA20P105_LC13_Input5		

Peak Table

Size [bp]	Calibrated Conc. [pg/µl]	Assigned Conc. [pg/µl]	Peak Molarity [pmol/l]	% Integrated Area	Peak Comment	Observations
25	446	-	27500	-		Lower Marker
328	6850	-	32100	100.00		
1500	250	250	256	-		Upper Marker

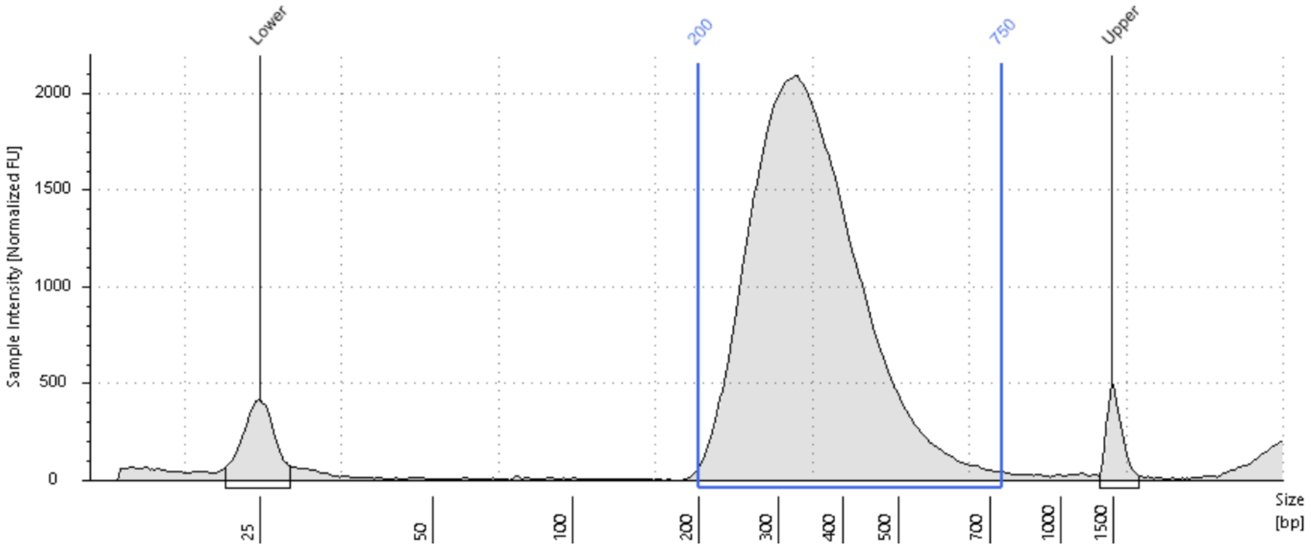

Region Table

From [bp]	To [bp]	Average Size [bp]	Conc. [pg/µl]	Region Molarity [pmol/l]	% of Total	Region Comment	Color
200	750	357	7750	35400	93.50		

© Agilent Technologies, Inc. 2021

F2: BEA20P105_LC14_Input6

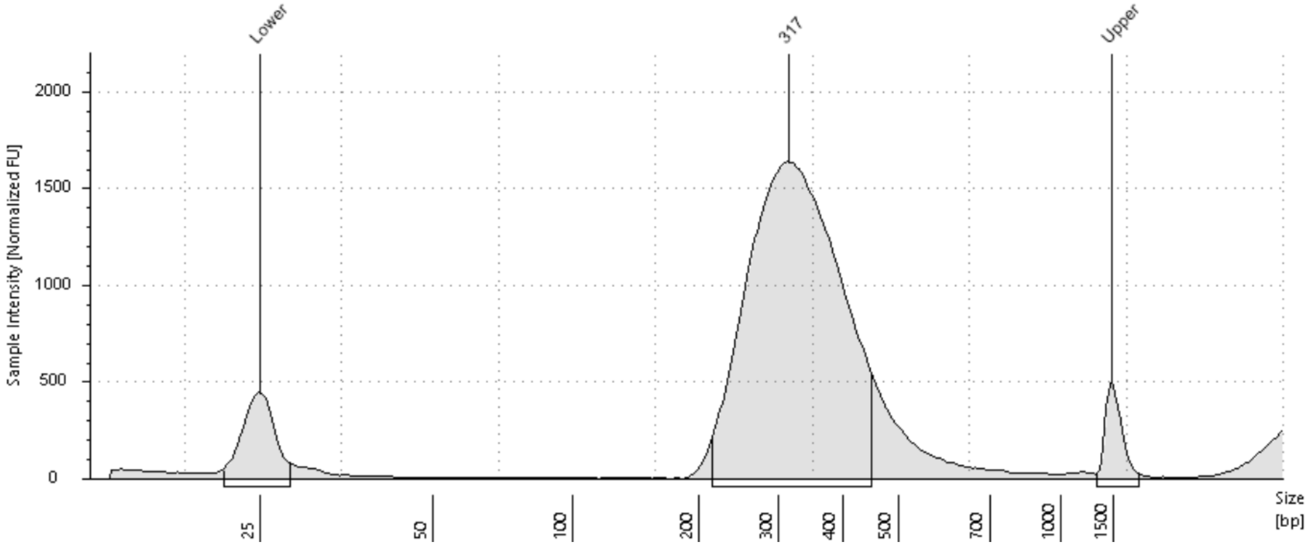

Sample Table

Well	Conc. [pg/µl]	Sample Description	Alert	Observations
F2	4770	BEA20P105_LC14_Input6		

Peak Table

Size [bp]	Calibrated Conc. [pg/µl]	Assigned Conc. [pg/µl]	Peak Molarity [pmol/l]	% Integrated Area	Peak Comment	Observations
25	445	-	27400	-		Lower Marker
317	4770	-	23200	100.00		
1500	250	250	256	-		Upper Marker

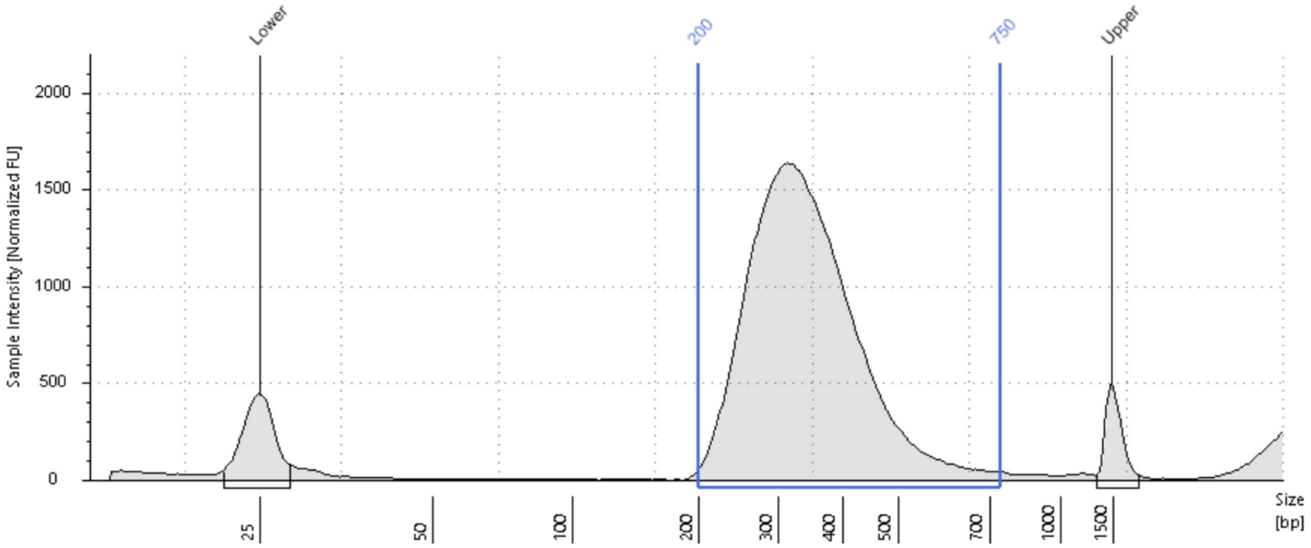

Region Table

From [bp]	To [bp]	Average Size [bp]	Conc. [pg/µl]	Region Molarity [pmol/l]	% of Total	Region Comment	Color
200	750	350	5360	24900	91.68		

© Agilent Technologies, Inc. 2021

G2: BEA20P105_LC15_Input7

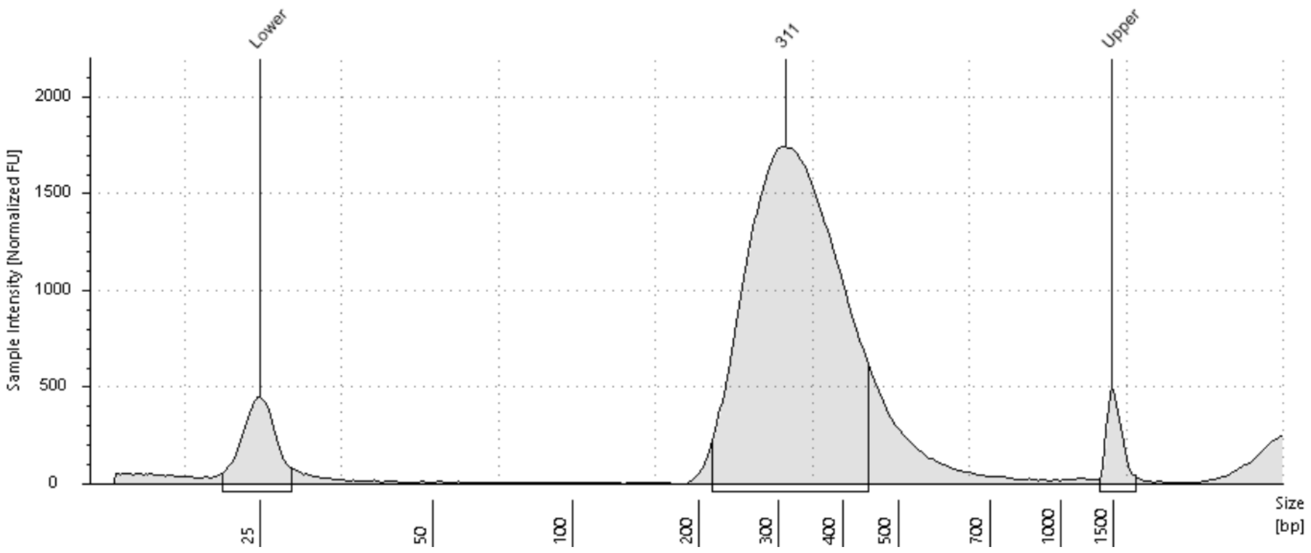

Sample Table

Well	Conc. [pg/µl]	Sample Description	Alert	Observations
G2	5610	BEA20P105_LC15_Input7		

Peak Table

Size [bp]	Calibrated Conc. [pg/µl]	Assigned Conc. [pg/µl]	Peak Molarity [pmol/l]	% Integrated Area	Peak Comment	Observations
25	509	-	31300	-		Lower Marker
311	5610	-	27800	100.00		
1500	250	250	256	-		Upper Marker

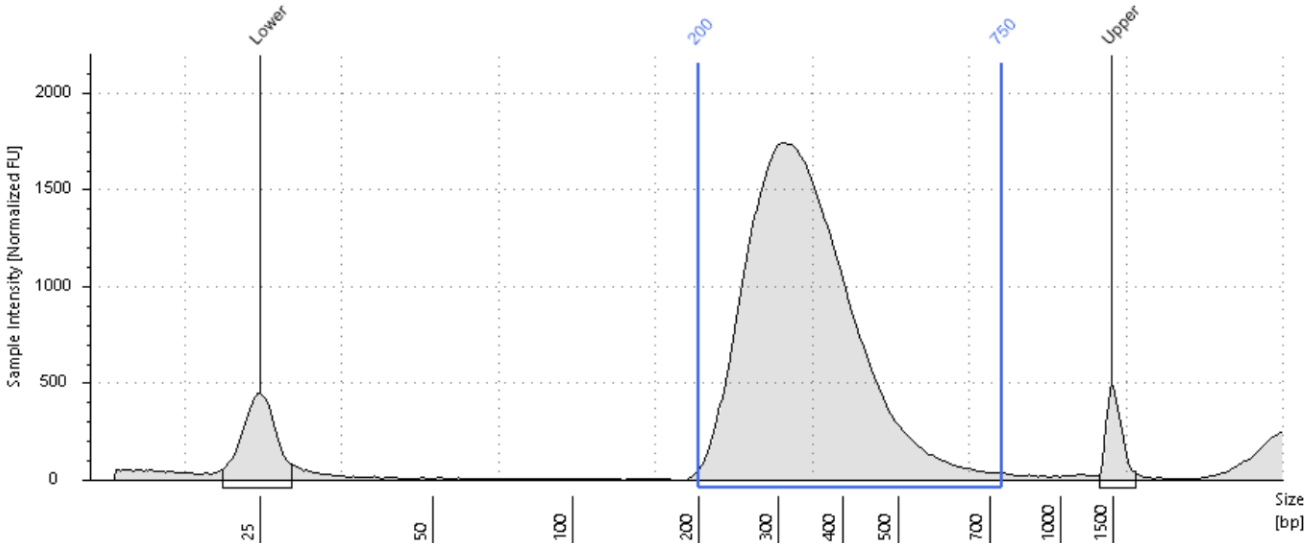

Region Table

From [bp]	To [bp]	Average Size [bp]	Conc. [pg/µl]	Region Molarity [pmol/l]	% of Total	Region Comment	Color
200	750	348	6360	29700	91.77		

© Agilent Technologies, Inc. 2021

H2: BEA20P105_LC16_Input8

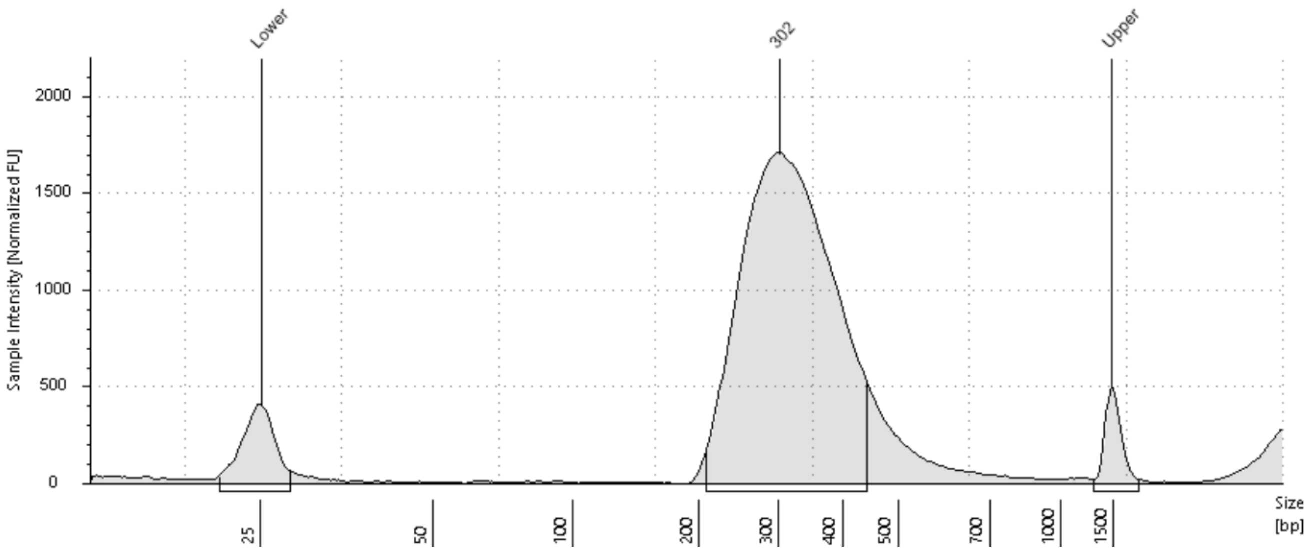

Sample Table

Well	Conc. [pg/µl]	Sample Description	Alert	Observations
H2	4980	BEA20P105_LC16_Input8		

Peak Table

Size [bp]	Calibrated Conc. [pg/µl]	Assigned Conc. [pg/µl]	Peak Molarity [pmol/l]	% Integrated Area	Peak Comment	Observations
25	411	-	25300	-		Lower Marker
302	4980	-	25400	100.00		
1500	250	250	256	-		Upper Marker

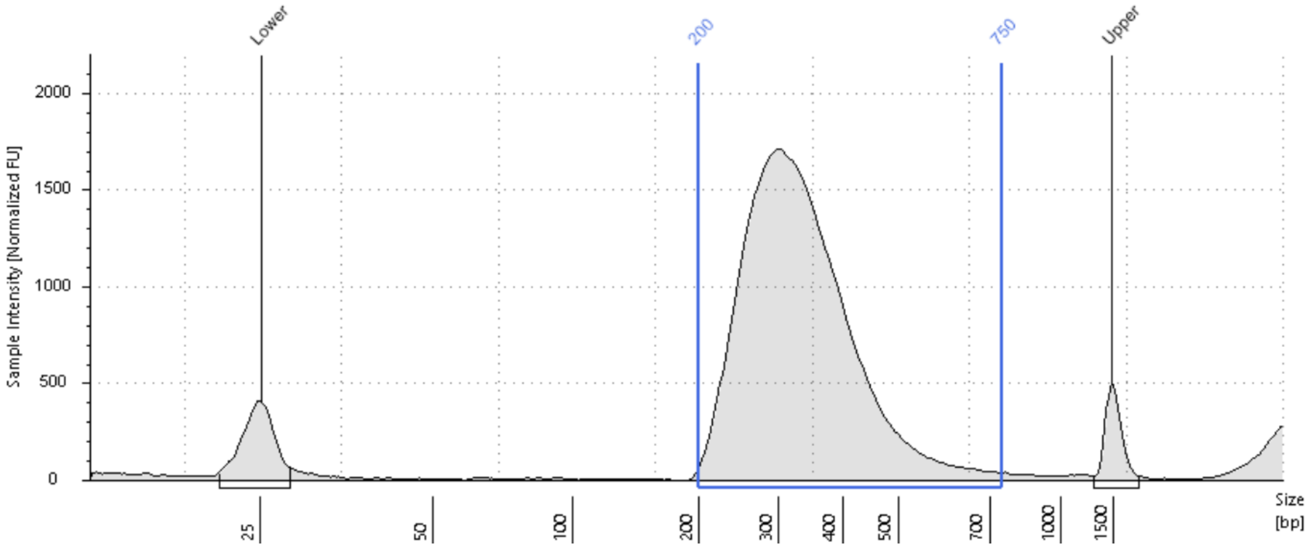

Region Table

From [bp]	To [bp]	Average Size [bp]	Conc. [pg/µl]	Region Molarity [pmol/l]	% of Total	Region Comment	Color
200	750	340	5560	26600	92.48		

© Agilent Technologies, Inc. 2021
